# Systematic Review and Model-Based Meta-Analysis of Targeted Drugs for Systemic Sclerosis

**DOI:** 10.3390/pharmaceutics18020250

**Published:** 2026-02-18

**Authors:** Marina Vaskeikina, Yaroslav Ugolkov, Boris Kireev, Kirill Peskov, Alina Volkova

**Affiliations:** 1 Faculty of Medicine, Lomonosov Moscow State University, 119991 Moscow, Russia; 2 Faculty of Bioengineering and Bioinformatics, Lomonosov Moscow State University, 119234 Moscow, Russia; yaroslav.ugolkov@msdecisions.tech (Y.U.); boris.kireev@msdecisions.tech (B.K.); 3 Modeling and Simulation Decisions LLC, Dubai 500767, United Arab Emirates; kirill.peskov@msdecisions.tech; 4 Marchuk Institute of Numerical Mathematics, Russian Academy of Sciences, 119991 Moscow, Russia; 5 Research Center of Model-Informed Drug Development, Sechenov First Moscow State Medical University, 119048 Moscow, Russia

**Keywords:** systemic sclerosis, targeted therapies, model-based meta-analysis, drug efficacy, comparative effectiveness, longitudinal modeling

## Abstract

**Background**: Systemic sclerosis (SSc) is a complex autoimmune fibrotic disorder marked by heterogeneous clinical features and multiple pathophysiological mechanisms. The rapid emergence of targeted therapies, aimed at selectively modulating molecular targets, has expanded treatment options; however, making direct efficacy comparisons remains challenging due to the variability in trial designs, endpoints, and patient populations. **Methods**: A systematic search of PubMed and ClinicalTrials.gov identified randomized controlled trials (RCTs) evaluating targeted therapies in SSc. A longitudinal mixed-effect meta-model incorporating Emax structural functions characterized treatment response trajectories for the modified Rodnan skin score (mRSS) and forced vital capacity (FVC). Between-study and between-treatment-arm variability were explicitly modeled to account for heterogeneity. **Results**: A total of 32 RCTs with 2036 patients and 23 targeted agents were analyzed. Guselkumab, an anti-IL-23 antibody, showed the greatest effect on mRSS, followed by tofacitinib, inebilizumab, and baricitinib. For FVC, B-cell-targeted therapies, with belimumab and rituximab, demonstrated the highest efficacy, while tocilizumab and nintedanib had more moderate effects. Time to 50% maximal response was approximately 27.5 weeks, indicating a 6.3-month period for half treatment response development. **Conclusions**: This model-based meta-analysis provides a broad comparison of targeted therapies in SSc, highlighting distinct efficacy patterns for skin versus lung involvement and offering hypothesis-generating insights that may support treatment selection and the design of future clinical trials.

## 1. Introduction

Systemic sclerosis (SSc) is a complex autoimmune disorder characterized by vascular injury and fibrosis across multiple organs, leading to a poor prognosis and a high risk of mortality [1,2,3]. Conventional therapies, including immunosuppressive drugs and autologous hematopoietic stem cell transplantation, provide some clinical benefits, but are constrained by serious adverse effects and inconsistent long-term disease control [4,5,6,7,8]. Among the most challenging manifestations are skin fibrosis and interstitial lung disease (ILD), which remain major contributors to functional impairment and mortality in SSc [9,10].

Recent advances in understanding SSc pathophysiology have enabled the development of targeted therapies that selectively modulate defined molecular targets (e.g., cytokines, surface receptors, intracellular kinases), in contrast to conventional immunosuppressants that exert broad, non-specific immune suppression [11,12,13]. A substantial number of targeted agents—117 clinical trials involving targeted therapies in SSc—were identified between January 2020 and April 2025 [14]. However, only three therapies—rituximab, tocilizumab, and nintedanib—have so far gained regulatory approval, with the latter two specifically indicated for SSc-associated ILD [15,16,17].

The substantial variability in study design, endpoint definition, and patient populations across trials, combined with small sample sizes, presents substantial challenges when attempting to compare efficacy indirectly across compounds [18]. Clinical trials in SSc use a broad range of endpoints to capture the disease’s heterogeneity [19]. Skin involvement in SSc is commonly assessed using the modified Rodnan skin score (mRSS), which measures skin fibrosis across 17 anatomical sites on a 0–3 scale, where higher scores indicate more severe skin thickening and disease progression [19,20,21]. Pulmonary involvement, a leading cause of mortality in SSc, is evaluated using lung function parameters such as forced vital capacity (FVC) and the diffusing capacity of the lungs for carbon monoxide (DLCO), which are usually expressed as percentages of predicted normal values. Reductions in FVC and DLCO indicate more advanced ILD and are associated with worse prognosis [19,22]. Additional endpoints include patient-reported outcomes (HAQ-DI, SHAQ), composite indices (ACR-CRISS), and assessments of vascular, gastrointestinal, cardiac, and renal involvement [19]. The diversity of these endpoints underscores the multi-organ nature of SSc and the challenges in assessing therapeutic efficacy across a heterogeneous patient population.

Due to the involvement of multiple pathophysiological pathways and organ systems in SSc, the success of clinical trials critically depends on the appropriate selection of clinical endpoints. For instance, in a Phase III trial with tocilizumab, no statistically significant improvement in skin fibrosis measured by primary endpoint mRSS was observed, but it significantly preserved lung function by reducing FVC decline, a secondary endpoint that supported its FDA approval for SSc-associated ILD [15,23,24].

Traditional meta-analytic approaches provide useful aggregate comparisons across studies but are fundamentally limited by their reliance on cross-sectional summaries at single timepoints. These methods cannot adequately account for the heterogeneity in follow-up duration, irregular measurement schedules, and sparse longitudinal structure that characterizes many SSc trials. As a result, potentially important determinants of between-study variability (BSV)—related to trial design, baseline severity, or mechanistic differences between interventions—may remain undetected.

Longitudinal model-based meta-analysis (MBMA) addresses several of these limitations by integrating the full-time course of response, pharmacological mechanisms, and covariate effects into a unified framework. This approach enables the interpolation and extrapolation of treatment effects to common timepoints, supports indirect comparisons across therapies, and allows for quantitative “what-if” simulations to explore how alternative designs, sample sizes, or patient subgroups might influence study outcomes. Recent applications of MBMA in other therapeutic areas have demonstrated its ability to identify complex sources of treatment-response variability and to improve trial design efficiency [25]. However, few existing MBMA applications have been tailored to the unique challenges of SSc, where longitudinal data are often sparse, observation times vary substantially across studies, and endpoints reflect diverse organ systems [26].

In the present study, we applied a longitudinal MBMA framework to quantify time-dependent treatment effects from published randomized controlled trials (RCTs) of targeted therapies in SSc. The developed model supports evidence-based decision-making by enabling indirect comparisons across compounds and informing optimal choices regarding trial duration and the timing of interim analyses, taking into account sample size, between-study and between-treatment-arm (BTAV) variability, and the dynamics of treatment response.

## 2. Materials and Methods

### 2.1. Study Eligibility and Data Collection

A systematic literature search was conducted using PubMed and ClinicalTrials.gov databases in accordance with PRISMA guidelines to identify RCT reporting efficacy scores in populations with SSc treated with targeted therapies [27]. This systematic review was registered on OSF on 22 January 2026 with the registration DOI number 10.17605/OSF.IO/NF3QK. Standard of care served as a comparator arm across studies. The PRISMA checklist is provided in the Supplementary Materials, Table S1. The search was conducted using the following terms applied within the databases: (disease-specific terms) AND (investigating targeted therapies) AND (publication type filters). The exact queries are available in the Supplementary Materials, Tables S2 and S3. Two authors independently screened all records for eligibility. Disagreements were resolved through discussion between the two reviewers. Records identified through systematic screenings were merged, and duplicates were then removed. If an abstract or summary was deemed valid for inclusion into the analysis, the original publication was investigated in detail and the relevant content was added to the database. Studies were eligible for inclusion if they satisfied the following criteria: (1) randomized controlled trial design; (2) enrollment of patients diagnosed with SSc; (3) reporting of clinical efficacy endpoint changes attributable to targeted therapeutic interventions; and (4) investigation of pharmacological agents with established mechanistic targets. The last update to the database was implemented on 20 October 2025. Reference management was performed using Zotero (7.0.32) software (https://www.zotero.org/ (accessed on 10 February 2026)).

### 2.2. Data Extraction

For data curation, a standardized Microsoft Excel database was developed with a predefined list of study design properties, population characteristics, and time-course clinical efficacy outcomes. The study design information included the type of targeted therapy, dose and dosing schedule for each treatment arm, study duration, and sample size. Population characteristics comprised SSc subtype and presence of ILD, sex distribution, age, race, baseline clinical endpoints (e.g., mRSS, FVC, DLCO), and biomarker profiles, including autoantibody positivity (e.g., anti-centromere status). Covariates were retained for analysis when missingness was below 20%; remaining missing values were imputed using population mean. The proportion of missing values for all covariates is available in Supplementary Materials, Table S4.

In addition, the change from baseline (CFB) for mRSS and FVC—two of the most frequently reported endpoints—were incorporated into the database. FVC values originally reported in milliliters were converted to percent predicted values (Equation (1)):

(1) $$FVC\;\%\;predicted=\;\frac{Absolute\;change\;in\;mL\;}{FVC\;in\;mL\;predicted\;}\times 100\%,$$

FVC predicted values (mL) were calculated using study-specific methodologies. In total, unit conversion from mL to percent predicted values was required for 3 of the 21 trials contributing FVC data. In the SENSCIS trial, both FVC in milliliters and FVC percent predicted values were reported at baseline for each arm, which allowed us to back-calculate arm-specific predicted FVC (3396 mL for the nintedanib arm and 3496 mL for the control arm) [28]. In the study by Allanore et al. (2020), predicted FVC was derived using the GLI-2022 race-neutral equations based on the reported distributions of sex, age, height, and predominant ethnicity in each arm, and these values were then used to express FVC as percent predicted values (2920 mL for the control arm and 2870 mL for the romilkimab arm) [29,30]. For the STRATUS study, the necessary anthropometric data were not available, so FVC percent predicted could not be calculated and this trial was excluded from the FVC modeling dataset [31].

When studies reported absolute endpoint values rather than CFB, all measurements were converted to CFB using Equation (2):

(2) $$CFB\left(t\right)=Y_{abs}\left(t\right)-Y_{BL},$$

where $Y_{abs}\left(t\right)$ is the absolute value of the endpoint at time t, $Y_{BL}$ is its corresponding baseline value.

Efficacy trajectories across studies were reported using different measures of central tendency (mean or median) and variability (SD, SE, IQR). All aggregated data were standardized to means and standard deviations (SDs) using published conversion Equations (3)–(6) [32,33]:

(3) $$Mean=\frac{Median+Q1+Q3}{3},$$

(4) $$SD=\frac{\left(Upper\;95\%CI-Lower\;95\%CI\right)\times \sqrt{N}}{3.92},$$

(5) $$SD=SE\times \sqrt{N},$$

(6) $$SD=\frac{Q3-Q1}{1.35},$$

where Q1 and Q3 denote the first and third quartiles, Upper 95%CI and Lower 95%CI indicate lower and upper bounds of the 95% confidence intervals (CI), N is the number of subjects in the arm, and SE is the standard error.

For treatment arms with fewer than 15 subjects, range-based (Min-Max) SD estimation was applied as recommended in small-sample settings (Equation (7)) [32,33]:

(7) $$SD=\sqrt{\frac{1}{12}\times {\left(\frac{Min-4\times Median+Max}{4}\right)}^{2}+{\left(Max-Min\right)}^{2}}$$

Data extraction from figures was performed using PlotDigitizer (3.1.6) (https://plotdigitizer.com (accessed on 10 February 2026)).

To explore potential publication bias, we constructed funnel plots and applied Egger’s regression test for both mRSS and FVC endpoints; the corresponding diagnostics are provided in Supplementary Materials, Figures S11 and S12, and demonstrate the absence of publication bias.

### 2.3. Longitudinal Nonlinear Mixed-Effect Model Development

#### 2.3.1. Structural Model

To characterize the time-course of two key efficacy endpoints, mRSS and FVC, a longitudinal MBMA framework was developed [34]. Several alternative structural models (linear, log-linear, an exponential approach-to-plateau model, sigmoidal Emax) were explored during model development. Based on goodness-of-fit diagnostics, AIC, and parameter identifiability, the Emax model provided the best description of the data and was therefore selected. Treatment-related CFB was described using Emax-type function in Equation (8):

(8) $$CFB_{ik}^{a}\left(t\right)=\;\frac{Emax_{ik}^{a}\times t}{ET50+t},$$

where $CFB_{ik}^{a}\left(t\right)$ is CFB over time for endpoint a in study i for k treatment arm, $Emax_{ik}^{a}$ is the arm-specific maximum treatment response, ET50 is the time to achieve 50% of maximum response, and t is the time in weeks.

A constant residual error weighted by sample size was used to describe residual variability (Equation (9)):

(9) $$CF{Bobs}_{ijk}^{a}=CFB_{ijk}^{a}+\varepsilon_{ijk}^{a},\;\varepsilon_{ijk}^{a}\sim N\left(0,\frac{\sigma_{a}^{2}}{N_{ijk}^{a}}\right),$$

where $CF{Bobs}_{ijk}^{a}$ and $CFB_{ijk}^{a}$ are the observed and predicted CFB for a outcome in study i at the time point j and treatment arm k, $\varepsilon_{ijk}^{a}$ is the constant residual error following a normal distribution with mean zero and variance $\sigma_{a}^{2}$ scaled by the number of subjects in the treatment arm $N_{ijk}^{a}$.

To capture the heterogeneity across studies and treatment arm, BSV and BTAV were incorporated on the Emax parameter (Equation (10)):

(10) $$Emax_{ik}^{a}=Emaxpop^{a}+{\eta_{Emax_{i}^{a}}}^{\left(0\right)}+{\eta_{Emax_{ik}^{a}}}^{\left(1\right)},\;{\eta_{Emax_{i}^{a}}}^{\left(0\right)}\sim N\left(0,\omega_{a}^{2}\right),\;{\eta_{Emax_{ik}^{a}}}^{\left(1\right)}\sim N\left(0,\frac{\gamma_{a}^{2}}{N_{ijk}^{a}}\right)$$

where $Emaxpop^{a}$ is the population parameter for Emax. The terms ${\eta_{Emax_{i}^{a}}}^{\left(0\right)}$ and ${\eta_{Emax_{ik}^{a}}}^{\left(1\right)}$ represent the BSV and BTAV following a normal distribution. The variance of BTAV $\gamma_{a}^{2}$ was scaled by sample size $N_{ijk}^{a}$ to reflect the fact that average responses in larger arms are estimated more precisely than in smaller arms. In this parameterization, BTAV represents noise in arm-level mean responses arising from inter-individual variability and finite sample sizes, and therefore decreases as $N_{ijk}^{a}$ increases, consistent with recent MBMA methodology recommendations [34]. The variance of BSV $\omega_{a}^{2}$ remained unweighted, reflecting heterogeneity driven by differences in inclusion criteria, geographical location, and study design, and is independent of recruitment magnitude.

#### 2.3.2. Covariate Model Building

Treatment effects were incorporated as a categorical covariate on Emax with the control arm serving as the reference category (Equation (11)):

(11) $$Emax_{ik}^{a}=Emaxpop^{a}+\sum \beta_{TRT}^{a}+\;{\eta_{Emax_{i}^{a}}}^{\left(0\right)}+{\eta_{Emax_{ik}^{a}}}^{\left(1\right)},$$

where $\beta_{TRT}^{a}$ is the effect of treatment TRT on Emax parameter.

Active treatment arms whose effects were estimated with insufficient precision, as indicated by a relative standard error (RSE) > 50% used as a heuristic criterion for acceptable parameter precision, were excluded from the model, while their corresponding control arms were retained to preserve informative control data. In addition to treatment effects, baseline covariates with less than 20% of missingness (specifically age, proportion of females, disease duration, baseline mRSS, and type of SSc) were explored as predictors of Emax. Continuous covariates were adjusted for the population median and introduced via a power function on Emax parameter; the categorical covariate was coded as indicator variable.

#### 2.3.3. Assessment of Model Performance

Model performance was evaluated based on changes in the objective function value, root mean square error (RMSE) value, diagnostic plots, and assessments of parameter precision. Diagnostic plots included observations versus individual predictions, individual weighted residuals versus time or predictions, and individual predictions over time overlaid with observations.

### 2.4. Model Simulations

To enable indirect comparison of treatment effects, a simulation-based framework was implemented. Model uncertainty was propagated by sampling parameter vectors from a multivariate normal distribution defined by the final parameter estimates and their corresponding variance–covariance matrix obtained during model calibration. For each treatment scenario, 1000 replicated simulations were generated. These simulated trajectories were then summarized to obtain mean predictions and the associated 95% CIs. The sample size was assumed to be infinite and between-study and between-treatment-arm variability (BSV/BTAV) were not included in these simulations in order to represent an idealized, theoretical setting in which all treatments are evaluated in the same standardized population in the absence of sampling noise. This approach was used to isolate structural treatment effects and to facilitate visual comparison across therapies, rather than to mimic realistic clinical trial conditions or predict observed variability.

To evaluate the impact of sample size on the precision of model predictions, an additional set of simulations was conducted using cohort sizes of 15, 45, and 288 patients. These values correspond to the median, mean, and maximum treatment-arm sample sizes observed in the curated dataset. For each cohort-size scenario, 1000 simulations were produced for the control (${CFB}_{control}^{a}$) and treatment (${CFB}_{treatment\;}^{a}$) arms while incorporating residual variability and BTAV (Equations (12) and (13)):

(12) $${CFB}_{control}^{a}\left(t\right)=\;\frac{({Emax}_{control}^{a}\;+\;{\eta_{Emax_{ik}^{a}control}}^{\left(1\right)})\;\times \;t}{ET50+t}\;+\varepsilon_{control\;}^{a},\;\varepsilon_{control}^{a}\sim N\left(0,\frac{\sigma_{a}^{2}}{N}\right),\;{\eta_{Emax_{ik}^{a}control}}^{\left(1\right)}\sim N\left(0,\frac{\gamma_{a}^{2}}{N}\right)$$

(13) $$\begin{array}{l} {CFB}_{treatment\;}^{a}\left(t\right)=\frac{{(Emax}_{control}^{a}+\beta_{treatment}+{\eta_{Emax_{ik}^{a}treatment}}^{\left(1\right)})\times t}{ET50+t}+\varepsilon_{treatment\;}^{a},\\ \varepsilon_{treatment}^{a}\sim N\left(0,\frac{\sigma_{a}^{2}}{N}\right),\;{\eta_{Emax_{ik}^{a}treatment}}^{\left(1\right)}\sim N\left(0,\frac{\gamma_{a}^{2}}{N}\right) \end{array}$$

The resulting simulated trajectories were used to derive mean profiles and corresponding 95% CIs for each endpoint and scenario.

To evaluate the earliest timepoint at which the treatment effect becomes statistically distinguishable from the control, at each timepoint $\left(t\right)$, the difference between paired simulated trajectories was computed (Equation (14)):

(14) $${\;\Delta \;}^{\left(i\right)}\left(t\right)=\;{CFB}_{treatment}^{a\left(i\right)}\left(t\right){\;-\;CFB}_{control}^{a\left(i\right)}\left(t\right),\;i=1,\ldots ,1000$$

A 95% CI for the difference in trajectories was then constructed from the 2.5th and 97.5th percentiles of ${\;\Delta \;}^{\left(i\right)}\left(t\right)$. The earliest timepoint at which this CI did not include zero was defined as the time to detectable treatment effect.

### 2.5. Software and Statistical Analysis

All statistical analyses, data handling, dataset formation for analysis, exploratory data analysis, and visualization were conducted using R Statistics version 4.4.2 (The R Foundation, Vienna, Austria). The packages used include readxl (1.4.3.), tidyverse (2.0.0.), patchwork (1.3.0.), cowplot (1.1.1), MASS (7.3.51.6), and forcats (1.0.0.). Parameter estimation was performed in Monolix (2024R1) using a nonlinear mixed-effects modeling approach implemented via the Stochastic Approximation Expectation–Maximization algorithm with linearization method to estimate log-likelihood.

## 3. Results

### 3.1. Study Selection, Patient Population, and Trial Characteristics

**Figure 1:**
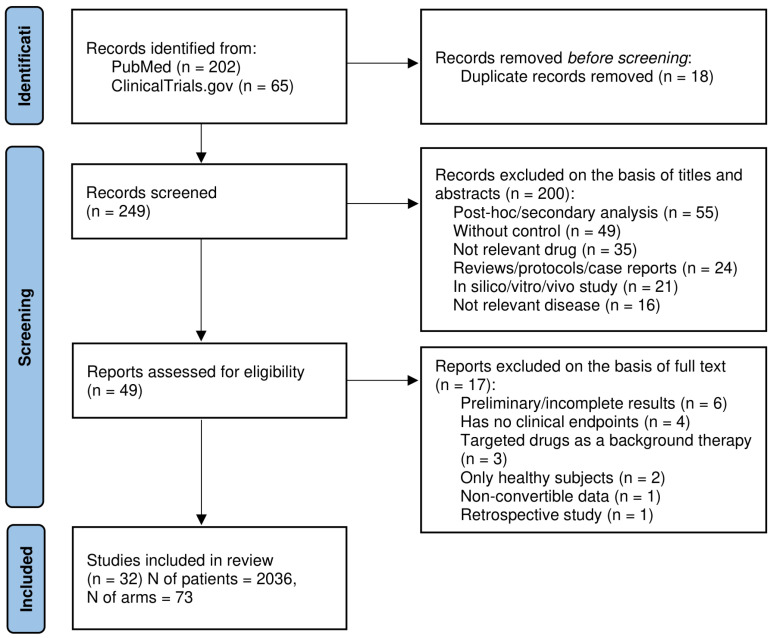
PRISMA flow diagram for search and selection of RCTs of targeted therapies in SSc.

The initial search of the PubMed and ClinicalTrials.gov databases yielded 267 potentially relevant records of RCTs in SSc. After removing 18 duplicates, 249 unique records remained for screening. Title and abstract evaluation resulted in the exclusion of 200 records due to post hoc or secondary analyses (55 studies); absence of a control group (49 studies); use of non-targeted therapies (35 studies); publication type, such as reviews, protocols, or case reports (24 studies); in silico, in vitro, or in vivo studies (21 studies); and studies addressing unrelated diseases (16 studies). A total of 49 full-text articles were evaluated for eligibility, of which 17 were excluded because of preliminary or incomplete results (6 studies), the absence of clinical efficacy endpoints (4 studies), the use of targeted agents as background therapy (3 studies), the inclusion of only healthy volunteers (2 studies), or retrospective design (1 study). One additional study (Acharya et al., 2020) reported mRSS as the median with the min-max range [35]. Due to the very small sample size and high response variability, the metric could not be reliably transformed into a mean, and the study was therefore excluded. Ultimately, 32 studies fulfilled all inclusion criteria and were incorporated into the exploratory analysis. The study selection flowchart is presented in Figure 1, and detailed study characteristics are provided in the Supplementary Materials, Table S5 [28,30,31,36,37,38,39,40,41,42,43,44,45,46,47,48,49,50,51,52,53,54,55,56,57,58,59,60,61,62,63,64].

The final dataset consisted of 73 study arms and 2036 patients (mean age 50.5 years; 75.5% women). The most common population was dcSSc, represented in 16 studies, with an additional 11 studies enrolling patients with SSc without specifying the subtype. One study included patients with SSc-PAH. Only 4 of the 32 studies explicitly reported the presence of ILD, although the lowest observed baseline FVC (60.3%) occurred in dcSSc cohorts. The mean baseline mRSS across all studies was 22.2, and the mean baseline percent predicted FVC was 83.4%.

**Figure 2:**
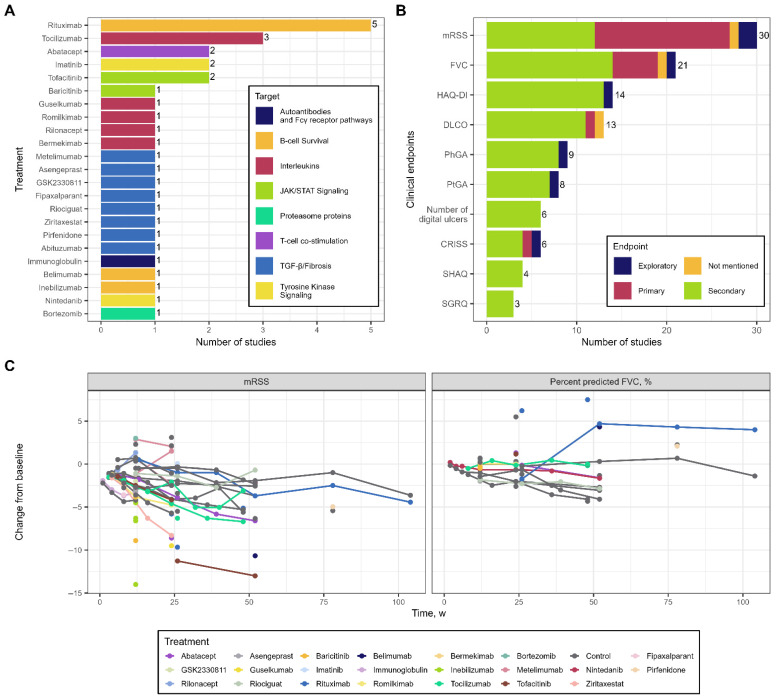
Exploratory data analysis of targeted therapies, clinical endpoints, and treatment responses in randomized controlled trials (RCTs) of systemic sclerosis (SSc). (**A**) Prevalence of targeted drugs evaluated in RCTs, grouped by their molecular targets. (**B**) Prevalence of clinical endpoints used in SSc trials, filtered to include only endpoints reported in more than two studies (count > 2). Abbreviations: mRSS, modified Rodnan skin score; FVC, forced vital capacity; HAQ-DI, Health Assessment Questionnaire–Disability Index; DLCO, diffusing capacity of the lungs for carbon monoxide; PhGA, Physician Global Assessment; PtGA, Patient Global Assessment; CRISS, Composite Response Index in Systemic Sclerosis; SHAQ, Scleroderma Health Assessment Questionnaire; SGRQ, St. George’s Respiratory Questionnaire. (**C**) Spaghetti plots of change from baseline for mRSS and FVC across all treatment arms; each line represents an individual trial arm.

Across the 32 trials, 23 targeted agents were evaluated. Eighteen therapies were tested in only a single clinical study, including all eight antifibrotic agents (Figure 2A). The largest evidence base was available for rituximab (five studies) and tocilizumab (three studies). Only one RCT was available for the third approved therapy, nintedanib. A detailed overview of the mechanisms of action is provided in Supplementary Materials, Table S6 [65,66,67,68,69,70,71,72,73,74,75,76].

The dataset contained 39 distinct clinical endpoints, although only 11 were reported in more than two trials. Nearly all studies (30 of 32) reported mRSS, which served as the primary endpoint in approximately half of the cases. FVC was the second most common endpoint, reported in 21 studies, but because it was available only in mL in three trials and anthropometric data allowed a valid conversion to percent predicted in only two of them, the final FVC analysis included 20 studies, including several that did not specifically enroll ILD populations but instead focused on dcSSc. Other endpoints included DLCO—like FVC, reflecting pulmonary functional status—along with patient-reported outcomes such as HAQ-DI, PhGA, and PtGA, though these were far less frequently measured (Figure 2B). Because mRSS and FVC were reported in more than half of all trials and frequently served as primary outcomes, these two endpoints were selected for longitudinal modeling. Consequently, the study of SSc-PAH [49], for which neither mRSS nor FVC was reported and for which the primary endpoint was 6MWD, was excluded from subsequent analyses.

Aggregated longitudinal trajectories for mRSS and FVC are shown in Figure 2C. Both endpoints exhibited substantial variability even within placebo arms, ranging from −6.3 to 3.1 units for mRSS and from −4.33% to 5.5% for FVC. Treatment durations varied widely across trials, with some studies including follow-up periods of up to two years, while others spanned only 2–3 months. Efficacy assessments were performed at markedly different time points across studies and treatment regimens, limiting the direct comparability of longitudinal profiles. Moreover, the majority of studies provided only a single post-baseline efficacy assessment (16 of 30 for mRSS and 14 of 20 for FVC), resulting in sparse time-course data that restricted the characterization of dynamic response patterns.

A total of 118 unique baseline characteristics were identified across the dataset; however, only the type of SSc, age, sex (percentage of females), disease duration, and baseline mRSS met the predefined threshold of <20% missingness and were therefore included in covariate analyses (Supplementary Materials, Table S4).

### 3.2. Model Development

The Emax model describing longitudinal mRSS and FVC trajectories was successfully developed and demonstrated a good overall fit to the data. The final MBMA model included 19 parameters (Table 1). Both BTAV and BSV were incorporated into the maximal CFB for each endpoint. Diagnostic plots showed no apparent bias in either treatment or control arms (Supplementary Materials, Figures S2 and S3). Supplementary Figures S4 and S5 illustrate the observed mRSS and FVC values alongside model-predicted trajectories for each trial arm, confirming that the model adequately captured the central trends and BSV and BTAV despite substantial heterogeneity in the underlying data.

**Figure 3:**
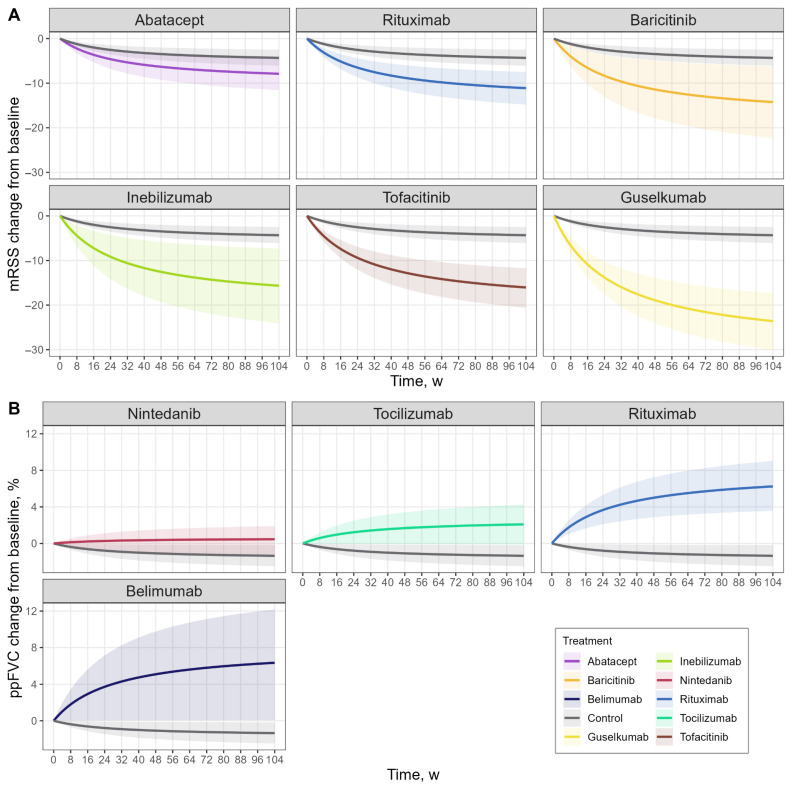
Model-predicted trajectories for therapies with robustly estimated differences from the control arm: (**A**) mRSS; (**B**) ppFVC. Colors indicate treatments, solid lines represent mean predictions, and shaded ribbons denote the corresponding 95% CI. ppFVC—percent predicted FVC.

In total, the estimated treatment effect for 14 therapies could not be distinguished from the reference group within the precision of the model. The estimated maximal effect for standard of care on mRSS was −5.29 points, reflecting an overall improvement in skin scores under background therapy. Six therapies showed clearly distinguishable effects relative to the standard of care, with varying levels of estimation precision driven by the number and size of contributing trials and the consistency of observed effects. The estimated effect on improvements in mRSS were as follows: guselkumab—anti-IL-23 antibody (−23.55 points, RSE 16.7%), tofacitinib—JAK1/JAK3 inhibitor (−14.35 points, RSE 18.4%), inebilizumab—anti-BAFF antibody (−13.45 points, RSE 38.0%), baricitinib—JAK1/JAK2 inhibitor (−13.87 points, RSE 35.4%), rituximab—anti-CD20 antibody (−8.03 points, RSE 26.3%), and abatacept—anti-CTLA-4 antibody (−5.41 points, RSE 44.6%) (Table 1, Figure 3A).

For FVC, an estimated maximal effect for standard of care was −2.29% predicted (RSE 35.3%), consistent with the progressive decline observed across most studies, and for 16 therapies, the modeled effect did not differ significantly from the reference group. Four therapies demonstrated model-estimated improvements in FVC that were clearly separated from the reference group: belimumab (+10.38%, RSE 43.0%), rituximab (+9.81%, RSE 20.1%), tocilizumab (+4.49%, RSE 31.7%), and nintedanib (+2.06%, RSE 45.5%) (Table 1, Figure 3B). Rituximab showed the highest precision due to contributions from four clinical trials, while the remaining therapies exhibited lower precision due to limited sample sizes or modest effect sizes.

Treatment rankings differed between endpoints: the largest mRSS effects were observed for agents targeting IL-23, JAK, and B-cell pathways, whereas the largest FVC effects were associated with B-cell-directed therapies; however, these patterns should be viewed as exploratory and interpreted with caution in light of sparse data and wide uncertainty intervals for several agents. The estimated time to half-maximal effect (*ET50*) was 27.49 weeks, indicating that approximately 6.3 months are required to achieve half of the maximal treatment response.

Covariate analyses evaluated SSc subtype, age, sex, disease duration, and baseline mRSS as potential predictors of Emax. None of the tested covariates improved model performance or reduced BSV. Therefore, no covariates were retained in the final model. Additionally, we explored covariate effects in an exploratory manner by correlating arm-specific estimates of Emax with relative covariate values, where available. This analysis did not reveal any meaningful or statistically robust correlations (Supplementary Materials, Figure S13).

### 3.3. Model Application

**Figure 4:**
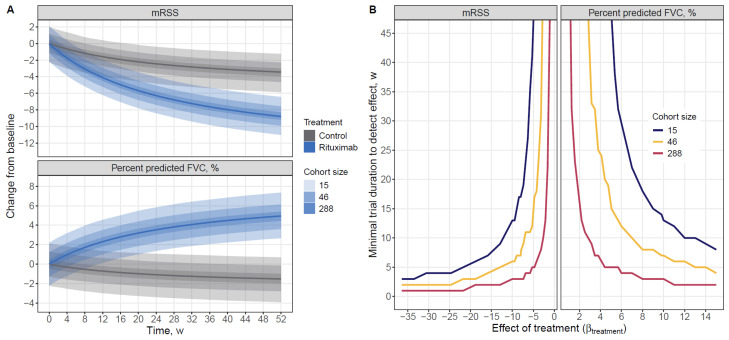
Influence of cohort size on model-based detection of treatment effects. (**A**) Colors represent treatment groups; solid lines denote mean predictions, and shaded ribbons indicate the corresponding 95% CIs, with ribbon opacity increasing proportionally to cohort size. (**B**) Colors indicate cohort size. Effect of treatment-value of $\beta_{treatment}$ model parameter.

To assess how cohort size influences BTAV in endpoint trajectories and the ability to detect treatment effects, we simulated longitudinal mRSS and FVC profiles for rituximab—which demonstrated clearly distinguishable effects relative to the control in both endpoints—and for the control arm. Figure 4A shows the predicted trajectories with corresponding 95% CIs for three representative sample sizes (*N* = 15, 46, and 288 per arm), reflecting the median, mean, and maximum treatment-arm sizes observed in the curated dataset. As expected, increasing the cohort size substantially reduced the width of the uncertainty bands, leading to progressively clearer visual separation between rituximab and control trajectories. For small cohorts (*N* = 15), the 95% CIs of the two arms overlapped for prolonged periods, whereas for large cohorts (*N* = 288), divergence became evident much earlier for both mRSS and FVC.

Figure 4B complements these findings by quantifying the minimal time point at which the treatment effect becomes statistically detectable across a range of assumed treatment-effect sizes (β_treatment_) for the same three sample sizes. Larger β_treatment_ values consistently resulted in earlier detection. However, the dependence of detection time on β_treatment_ differed markedly by cohort size: large cohorts showed both shorter overall detection times and a steeper decline in detection time as β_treatment_ increased, indicating high sensitivity to even moderate effect sizes. In contrast, small cohorts exhibited delayed detectability unless the effect size was substantial, and the reduction in detection time with increasing β_treatment_ was comparatively modest.

When applying this framework to rituximab (β_Rituximab_ = −8.03 for mRSS and 9.81 for FVC), the minimal trial duration varied considerably across sample sizes. For mRSS, statistically significant differences were detectable as early as 3 weeks for *N* = 288, compared with 17 weeks for *N* = 15. For FVC, the corresponding detection times ranged from 3 to 14 weeks across the same sample-size scenarios.

## 4. Discussion

This study presents the first MBMA describing the longitudinal trajectories of the two core SSc endpoints—mRSS and FVC—using an Emax time-course framework to quantify treatment effects across all targeted therapies investigated in RCTs. This systematic review and model-based meta-analysis was conducted in accordance with PRISMA recommendations and was registered in OSF. The search was restricted to PubMed and ClinicalTrials.gov and did not include subscription-based databases such as Embase, Web of Science, or Cochrane CENTRAL, which is an important limitation, and may have resulted in incomplete retrieval of eligible RCTs. To mitigate this risk, we manually screened reference lists of all included trials and key systematic reviews and meta-analyses in SSc and SSc-ILD to reduce the likelihood that major targeted therapies or pivotal trials were missed.

To construct the model, we assembled a comprehensive database comprising 118 baseline and 39 longitudinal datapoints extracted from prospective RCTs. Only mRSS and FVC were modeled, as these were the only endpoints consistently reported in at least half of all trials; moreover, FVC was harmonized across studies by converting reported values in milliliters to % predicted, allowing its treatment as a unified pulmonary endpoint. Sensitivity analysis excluding trials requiring this unit conversion showed that all key model parameters changed by at most 20% and the set of therapies with distinguishable effect on FVC remained unchanged. Despite the breadth of available data, only five baseline characteristics (SSc subtype, age, sex, disease duration, and baseline mRSS) met the threshold for covariate analysis (missingness < 20%), illustrating the substantial heterogeneity in current SSc trials in terms of reported biomarkers.

Longitudinal MBMA has not previously been applied to SSc. A recent network meta-analysis by Wu et al. evaluated treatments for SSc-ILD using a shared-placebo comparator and demonstrated that tocilizumab had the highest probability of improving FVC, while rituximab most strongly affected DLCO [77]. Additional meta-analyses have focused on preclinical effects of JAK inhibitors in murine models [67], or on individual interventions such as mycophenolate mofetil, cyclophosphamide, and autologous hematopoietic stem cell transplantation [78,79,80,81]. Collectively, the existing evidence syntheses tend to target narrow therapeutic or phenotypic subsets, rather than enabling a unified comparative assessment across all targeted therapies. To highlight the added value of MBMA, we performed a classical meta-analysis using only the final reported study timepoints. While overall conclusions were aligned, MBMA identified significant treatment effects for baricitinib and abatacept on mRSS and for belimumab on FVC, effects that were not detectable via traditional meta-analysis (Figures S8 and S9). This discrepancy likely reflects the ability of MBMA to leverage longitudinal data and explicitly model the time-course of drug response.

The Emax time-course framework was selected for its physiological plausibility and interpretability within a clinical context, consistent with prior work in chronic inflammatory diseases such as rheumatoid arthritis [82]. Alternative approaches—such as exponential saturation models or log-linear forms—have also been applied successfully in other therapeutic areas (e.g., [83]), but in the current analysis they provided poorer fits, with higher AIC values, less stable parameter estimates, and less adequate capture of the observed trajectories than the Emax specification. A shared ET50 parameter was estimated for both endpoints under the assumption of a common time to achieve half-maximal response; given the larger volume of mRSS data, skin assessments contributed more strongly to ET50 estimation than pulmonary assessments. A model variant with endpoint-specific ET50 values was explored, but for FVC, this resulted in an implausibly long ET50 of 77.61 weeks and an almost linear trajectory, driven by the very sparse data beyond week 52 (only three placebo observations). To avoid this structural identifiability issue, we retained a single ET50, which provided a more robust and parsimonious description of both mRSS and FVC trajectories and was consistent with the observed inflection in their longitudinal profiles, and supported by the adequacy of model diagnostics (Supplementary Figures S2–S7).

Endpoint-specific Emax BSV and BTAV parameters were estimated independently, which is appropriate given the weak correlation between changes in mRSS and FVC (Supplementary Materials, Figure S10, r = −0.33), indicating that improvements in skin fibrosis only modestly parallel improvements in lung function. Joint modeling via latent variables, which is commonly used when endpoints reflect a shared underlying disease process, was therefore not pursued because the weak correlation would likely overcomplicate the model without improving predictive power [84].

Across therapies, guselkumab demonstrated the largest effect on mRSS. This finding is mechanistically plausible given the central role of IL-23 in promoting Th17 differentiation and IL-17-mediated fibroinflammatory signaling in SSc [85]. JAK inhibitors—including tofacitinib and baricitinib—also demonstrated pronounced effects on mRSS, consistent with increasing evidence implicating JAK/STAT activation in fibroblast proliferation and immune dysregulation in SSc [67,86]. For pulmonary outcomes, belimumab and rituximab produced the largest model-estimated improvements in FVC, exceeding those of approved agents such as nintedanib and tocilizumab. Both drugs modulate B-cell biology—belimumab via BAFF neutralization and rituximab via CD20 depletion [66]. Belimumab did not demonstrate statistically significant FVC improvement in its RCT [38], but an effect emerged in the MBMA, albeit with low precision. However, these comparative inferences should be interpreted cautiously, as they are influenced by sparse longitudinal data, single-trial contributions for several agents, and wide confidence intervals.

Notably, no antifibrotic drug showed significant benefit for either mRSS or FVC in the MBMA. Although small, statistically significant antifibrotic effects have been reported in individual trials [62], the model-estimated maximum effects of metelimumab, pirfenidone, and related therapies remained comparable to standard therapy when assessed longitudinally, suggesting that immunomodulation rather than antifibrotic targeting may be required to meaningfully alter the course of established fibrosis.

The MBMA estimates average effects across all dose arms rather than modeling dose–response relationships explicitly. Of all therapies, only metelimumab and inebilizumab had sufficient dose-ranging data, yet neither demonstrated a clear increase in efficacy with higher dosing, reducing the risk of systematic bias from dose pooling.

Several implications for clinical trial design and model-informed drug development arise from our findings. First, the simulations demonstrate that the time at which statistically significant separation between treatment and control becomes detectable is determined jointly by sample size and the magnitude of the treatment effect. Even for a therapy with a moderate effect size, such as rituximab, detectable divergence from control occurred only after approximately 17 weeks when cohort size was small (*N* = 15). Given that most SSc trials schedule their primary assessments between 12 and 24 weeks, these results suggest that the lower end of this window may be insufficient for reliably detecting treatment effects in modestly sized cohorts, potentially leading to underpowered conclusions even when true differences exist.

Second, although MBMA has the potential to quantify sources of between-trial heterogeneity and facilitate virtual control generation, we were unable to identify statistically robust covariate drivers despite substantial BSV. This limitation aligns with broader observations that summary-level datasets often lack key prognostic and mechanistic variables—such as autoantibody profiles, baseline activity biomarkers, treatment history, which limits the ability to capture true response variability [87]. The inability to detect covariate effects in our meta-dataset underscores future research priorities. Integration of individual patient data, standardized reporting of key prognostic covariates (including autoantibody profiles, baseline severity metrics, prior treatment exposure, and relevant biomarkers), and development of joint models linking skin and lung endpoints would strengthen the capacity to explain heterogeneity and to support personalized, model-informed trial designs.

Finally, the predictive capabilities of the model directly support model-informed adaptive trial designs. By projecting the expected timing and magnitude of arm separation conditional on sample size, follow-up duration, and assumed effect sizes, the model provides quantitative inputs that can be incorporated into adaptive decision rules—for example, futility assessments, adaptive sample-size re-estimation, or conditional extension of follow-up when interim data suggest delayed treatment divergence [88]. Although MBMA is not itself a Bayesian adaptive framework, it supplies the prior results, predictive trajectories, and variance estimates that form the basis for Bayesian model-assisted design optimization [89]. Such applications are particularly valuable in rare diseases such as SSc, where recruitment constraints and endpoint BSV necessitate efficient, data-sparing, and mechanistically informed strategies for trial planning.

## 5. Conclusions

This study provides a broad, model-based comparison of targeted therapies in SSc, summarizing their longitudinal effects on skin and lung endpoints within a unified Emax framework. The key findings include the following: 1. Mechanistic selectivity emerges as a dominant pattern: IL-23 antagonism and JAK inhibition predominantly benefit skin manifestations, while B-cell depletion strategies show marked superiority for pulmonary fibrosis preservation. This divergence suggests distinct mechanistic drivers of cutaneous versus pulmonary disease progression. 2. Guselkumab shows the largest modeled effect for skin fibrosis (−23.55 mRSS points) with relatively high precision, supported by well-powered clinical trial data where available. 3. B-cell-depleting agents (rituximab and belimumab) demonstrate potential FVC benefits, indicating particular relevance for SSc-associated interstitial lung disease. 4. Substantial heterogeneity in current SSc trial methodology (118 baseline characteristics, 39 endpoints) with limited standardization constrains evidence synthesis and highlights the urgent need for community consensus on endpoint definitions and assessment protocols. 5. The MBMA framework provides a generalizable approach for model-informed adaptive trial design, enabling more efficient trial conduct through early efficacy prediction and scenario simulation. These findings should be interpreted as hypothesis-generating and as a basis for prioritizing future comparative and mechanistic studies. The analytical framework established herein is readily adaptable to future SSc trials as additional evidence accumulates, enabling iterative refinement of treatment efficacy estimates and therapeutic recommendations.

## Figures and Tables

**Table 1:** Parameter estimates of final longitudinal MBMA model.

Parameter	Value	RSE (%)
Maximum change in mRSS of the comparator ($Emaxpop^{mRSS}$)	−5.29	22.2
Effect of abatacept on mRSS reduction ($\beta_{Abatacept}^{mRSS}$)	−5.41	44.6
Effect of baricitinib on mRSS reduction ($\beta_{Baricitinib}^{mRSS}$)	−13.87	35.4
Effect of guselkumab on mRSS reduction ($\beta_{Guselkumab}^{mRSS}$)	−23.55	16.7
Effect of inebilizumab on mRSS reduction ($\beta_{Inebilizumab}^{mRSS}$)	−13.45	38.0
Effect of rituximab on mRSS reduction ($\beta_{Rituximab}^{mRSS}$)	−8.03	26.3
Effect of tofacitinib on mRSS reduction ($\beta_{Tofacitinib}^{mRSS}$)	−14.35	18.4
Standard deviation of BSV for change in mRSS ($\omega_{Emax}^{mRSS}$)	4.53	19.6
Standard deviation of BTAV for change in mRSS ($\gamma_{Emax}^{mRSS}$)	10.23	28.7
Standard deviation of residual error for change in mRSS ($\sigma^{mRSS}$)	4.12	10.0
Maximum change in FVC of the comparator in % ($Emaxpop^{FVC}$)	−2.29	35.3
Effect of nintedanib on FVC improvement ($\beta_{Nintedanib}^{FVC}$)	2.06	45.5
Effect of rituximab on FVC improvement ($\beta_{Rituximab}^{FVC}$)	9.81	20.1
Effect of tocilizumab on FVC improvement ($\beta_{Tocilizumab}^{FVC}$)	4.49	31.7
Effect of belimumab on FVC improvement ($\beta_{Belimumab}^{FVC}$)	10.38	43.0
Standard deviation of BSV for change in FVC ($\omega_{Emax}^{FVC}$)	1.97	38.0
Standard deviation of BTAV for change in FVC ($\gamma_{Emax}^{FVC}$)	9.94	30.1
Standard deviation of residual error for change in FVC ($\sigma^{FVC}$)	4.42	12.0
Time to 50% maximum change in mRSS and FVC in weeks (ET50)	27.49	16.6

## Data Availability

No new data were created or analyzed in this study.

## Supplementary Materials

The following supporting information can be downloaded at https://www.mdpi.com/article/10.3390/pharmaceutics18020250/s1, Table S1: PRISMA 2020 checklist; Table S2: Final query for search in PubMed; Table S3: Final query for search in ClinicalTrials.gov database; Table S4: Summary statistics of the covariates; Table S5: Summary of selected trials; Table S6: Drug targets; Figure S1: Prevalence of baseline characteristics in clinical trials; Figure S2: Individual fits for SSc endpoint mRSS per studies; Figure S3: Individual fits for SSc endpoint FVC per studies; Figure S4: Observed vs. predicted values for mRSS; Figure S5: Observed vs. predicted values for FVC; Figure S6: Weighted residual distribution from time and predicted values for mRSS score; Figure S7: Weighted residual distribution from time and predicted values for FVC score; Figure S8: Meta-analysis results for mRSS using final study timepoint; Figure S9: Meta-analysis results for FVC using final study timepoint; Figure S10: Correlation between changes in mRSS and FVC; Figure S11: Funnel plot assessing publication bias in SMD of mRSS; Figure S12: Funnel plot assessing publication bias in SMD of FVC; Figure S13: Correlation of individual parameter estimates with baseline covariates.

## Abbreviations

The following abbreviations are used in this manuscript:

SSc	Systemic sclerosis
mRSS	Modified Rodnan skin score
FVC	Forced vital capacity
DLCO	Diffusing capacity of the lungs for carbon monoxide
MBMA	Model-based meta-analysis
RCTs	Randomized controlled trials
ILD	Interstitial lung disease
HAQ-DI	Health Assessment Questionnaire–Disability Index
SHAQ	Scleroderma Health Assessment Questionnaire
PhGA	Physician Global Assessment
PtGA	Patient Global Assessment
ACR-CRISS	American College of Rheumatology Composite Response Index in Systemic Sclerosis
PAH	Pulmonary arterial hypertension
6MWD	Six-minute walk distance
CFB	Change from baseline
SD	Standard deviation
RSE	Relative standard error
BSV	Between-study variability
BTAV	Between-treatment-arm variability
Emax	Maximum treatment response
ET50	Time to 50% maximum response
IL	Interleukin
JAK	Janus kinase
PRISMA	Preferred Reporting Items for Systematic Reviews and Meta-Analyses
BAFF	B-cell-activating factor
