# Supplementary material for: Systematic Review and Model-Based Meta-Analysis of Targeted Drugs for Systemic Sclerosis
Source: Pharmaceutics. 2026 Feb 18;18(2):250. doi: 10.3390/pharmaceutics18020250 (PMC12944528; doi:10.3390/pharmaceutics18020250)
Supplement: Supplementary file 1 [file pharmaceutics-18-00250-s001.zip › pharmaceutics-4070676-supplementary.pdf]

Supplementary Materials to the manuscript:

Systematic Review and Model-Based Meta-Analysis of Targeted Drugs for Systemic Sclerosis

Marina Yu. Vaskeikina [1,*], Yaroslav A. Ugolkov [2,3,4], Boris V. Kireev [2,3,4], Kirill V. Peskov [3,4,5] and Alina A. Volkova [3,4,*]

[1] Faculty of Medicine, Lomonosov Moscow State University, 119991 Moscow, Russia
[2] Faculty of Bioengineering and Bioinformatics, Lomonosov Moscow State University, 119234 Moscow, Russia; yaroslav.ugolkov@msdecisions.tech (Y. U.); boris.kireev@msdecisions.tech (B. K.)
[3] Modeling and Simulation Decisions LLC, 500767, Dubai, United Arab Emirates; kirill.peskov@msdecisions.tech (K. P.)
[4] Marchuk Institute of Numerical Mathematics, Russian Academy of Sciences, Moscow, Russia
[5] Research Center of Model-Informed Drug Development, I.M. Sechenov First Moscow State Medical Uni-versity, Ministry of Health of Russia, 119048, Moscow, Russia;
* Correspondence: marina.vaskeykina@student.msu.ru (M. V.); alina.volkova@msdecisions.tech (A. V.)

Table of Contents

- Table S1: PRISMA 2020 checklist
- Table S2: Final query for search in PubMed
- Table S3: Final query for search in ClinicalTrials.gov database
- Table S4: Summary statistics of the covariates
- Table S5: Summary of selected trials
- Table S6: Drug targets
- Figure S1: Prevalence of baseline characteristics in clinical trials
- Figure S2: Individual fits for SSc end point mRSS per studies
- Figure S3: Individual fits for SSc end point FVC per studies
- Figure S4: Observed vs Predicted values for mRSS
- Figure S5: Observed vs Predicted values for FVC
- Figure S6: Weighted residuals distribution from time and predicted values for mRSS score
- Figure S7: Weighted residuals distribution from time and predicted values for FVC score
- Figure S8: Meta-analysis results for mRSS using final study timepoint
- Figure S9: Meta-analysis results for FVC using final study timepoint
- Figure S10: Correlation between changes in mRSS and FVC
- Figure S11: Funnel plot assessing publication bias in SMD of mRSS
- Figure S12: Funnel plot assessing publication bias in SMD of FVC
- Figure S13: Correlation of individual parameter estimates with baseline covariates.
- References

Table S1. PRISMA checklist

PRISMA 2020 Main Checklist

Topic	No.	Item	Location where item is reported
**TITLE**			
**Title**	1	Identify the report as a systematic review.	Title
**ABSTRACT**			
**Abstract**	2	See the PRISMA 2020 for Abstracts checklist	
**INTRODUCTION**			
**Rationale**	3	Describe the rationale for the review in the context of existing knowledge.	Section 1
**Objectives**	4	Provide an explicit statement of the objective(s) or question(s) the review addresses.	Section 1
**METHODS**			
**Eligibility criteria**	5	Specify the inclusion and exclusion criteria for the review and how studies were grouped for the syntheses.	Section 2.1
**Information sources**	6	Specify all databases, registers, websites, organisations, reference lists and other sources searched or consulted to identify studies. Specify the date when each source was last searched or consulted.	Section 2.1
**Search strategy**	7	Present the full search strategies for all databases, registers and websites, including any filters and limits used.	Table S2, S3
**Selection process**	8	Specify the methods used to decide whether a study met the inclusion criteria of the review, including how many reviewers screened each record and each report retrieved, whether they worked independently, and if applicable, details of automation tools used in the process.	Section 2.1
**Data collection process**	9	Specify the methods used to collect data from reports, including how many reviewers collected data from each report, whether they worked independently, any processes for obtaining or confirming data from study investigators, and if applicable, details of automation tools used in the process.	Section 2.2
**Data items**	10a	List and define all outcomes for which data were sought. Specify whether all results that were compatible with each outcome domain in each study were sought (e.g. for all measures, time points, analyses), and if not, the methods used to decide which results to collect.	Section 2.2
	10b	List and define all other variables for which data were sought (e.g. participant and intervention characteristics, funding sources). Describe any assumptions made about any missing or unclear information.	Section 2.2
**Study risk of bias assessment**	11	Specify the methods used to assess risk of bias in the included studies, including details of the tool(s) used, how many reviewers assessed each study and whether they worked independently, and if applicable, details of automation tools used in the process.	Section 2.1
**Effect measures**	12	Specify for each outcome the effect measure(s) (e.g. risk ratio, mean difference) used in the synthesis or presentation of results.	Section 2.3
**Synthesis methods**	13a	Describe the processes used to decide which studies were eligible for each synthesis (e.g. tabulating the study intervention characteristics and comparing against the planned groups for each synthesis (item 5)).	N/A

	13b	Describe any methods required to prepare the data for presentation or synthesis, such as handling of missing summary statistics, or data conversions.	Section 2.2
	13c	Describe any methods used to tabulate or visually display results of individual studies and syntheses.	Section 2.2
	13d	Describe any methods used to synthesize results and provide a rationale for the choice(s). If meta-analysis was performed, describe the model(s), method(s) to identify the presence and extent of statistical heterogeneity, and software package(s) used.	Section 2.3, Section 2.4, Section 2.5
	13e	Describe any methods used to explore possible causes of heterogeneity among study results (e.g. subgroup analysis, meta-regression).	Section 2.3.2
	13f	Describe any sensitivity analyses conducted to assess robustness of the synthesized results.	Section 2.4
**Reporting bias assessment**	14	Describe any methods used to assess risk of bias due to missing results in a synthesis (arising from reporting biases).	Section 2.2.
**Certainty assessment**	15	Describe any methods used to assess certainty (or confidence) in the body of evidence for an outcome.	N/A
**RESULTS**			
**Study selection**	16a	Describe the results of the search and selection process, from the number of records identified in the search to the number of studies included in the review, ideally using a flow diagram.	Figure 1
	16b	Cite studies that might appear to meet the inclusion criteria, but which were excluded, and explain why they were excluded.	Section 3.1
**Study characteristics**	17	Cite each included study and present its characteristics.	Table S5
**Risk of bias in studies**	18	Present assessments of risk of bias for each included study.	Figures S10, S11
**Results of individual studies**	19	For all outcomes, present, for each study: (a) summary statistics for each group (where appropriate) and (b) an effect estimate and its precision (e.g. confidence/credible interval), ideally using structured tables or plots.	Table S5, Figure S2, Figure S3
**Results of syntheses**	20a	For each synthesis, briefly summarise the characteristics and risk of bias among contributing studies.	Section 3.2
	20b	Present results of all statistical syntheses conducted. If meta-analysis was done, present for each the summary estimate and its precision (e.g. confidence/credible interval) and measures of statistical heterogeneity. If comparing groups, describe the direction of the effect.	Section 3.2
	20c	Present results of all investigations of possible causes of heterogeneity among study results.	Section 3.2
	20d	Present results of all sensitivity analyses conducted to assess the robustness of the synthesized results.	Section 3.3, Figures S8–S9
**Reporting biases**	21	Present assessments of risk of bias due to missing results (arising from reporting biases) for each synthesis assessed.	Figures S10, S11
**Certainty of evidence**	22	Present assessments of certainty (or confidence) in the body of evidence for each outcome assessed.	N/A
**DISCUSSION**			
**Discussion**	23a	Provide a general interpretation of the results in the context of other evidence.	Section 4
	23b	Discuss any limitations of the evidence included in the review.	Section 4
	23c	Discuss any limitations of the review processes used.	Section 4
	23d	Discuss implications of the results for practice, policy, and future research.	Section 4, Section 5

**OTHER INFORMATION**			
**Registration and protocol**	24a	Provide registration information for the review, including register name and registration number, or state that the review was not registered.	Materials and Methods 2.1
	24b	Indicate where the review protocol can be accessed, or state that a protocol was not prepared.	Materials and Methods 2.1
	24c	Describe and explain any amendments to information provided at registration or in the protocol.	N/A
**Support**	25	Describe sources of financial or non-financial support for the review, and the role of the funders or sponsors in the review.	Sections Funding, Acknowledgements
**Competing interests**	26	Declare any competing interests of review authors.	Section Conflict of interest
**Availability of data, code and other materials**	27	Report which of the following are publicly available and where they can be found: template data collection forms; data extracted from included studies; data used for all analyses; analytic code; any other materials used in the review.	Section Data availability statement

PRIMSA Abstract Checklist

Topic	No.	Item	Reported?
**TITLE**			
**Title**	1	Identify the report as a systematic review.	Yes
**BACKGROUND**			
**Objectives**	2	Provide an explicit statement of the main objective(s) or question(s) the review addresses.	Yes
**METHODS**			
**Eligibility criteria**	3	Specify the inclusion and exclusion criteria for the review.	Yes
**Information sources**	4	Specify the information sources (e.g. databases, registers) used to identify studies and the date when each was last searched.	Yes
**Risk of bias**	5	Specify the methods used to assess risk of bias in the included studies.	Yes
**Synthesis of results**	6	Specify the methods used to present and synthesize results.	Yes
**RESULTS**			
**Included studies**	7	Give the total number of included studies and participants and summarise relevant characteristics of studies.	Yes
**Synthesis of results**	8	Present results for main outcomes, preferably indicating the number of included studies and participants for each. If meta-analysis was done, report the summary estimate and confidence/credible interval. If comparing groups, indicate the direction of the effect (i.e. which group is favoured).	Yes
**DISCUSSION**			
**Limitations of evidence**	9	Provide a brief summary of the limitations of the evidence included in the review (e.g. study risk of bias, inconsistency and imprecision).	Yes
**Interpretation**	10	Provide a general interpretation of the results and important implications.	Yes
**OTHER**			
**Funding**	11	Specify the primary source of funding for the review.	Yes
**Registration**	12	Provide the register name and registration number.	Yes

Table S2. Final query for search in PubMed.

(scleroderma, systemic[MeSH Terms] OR scleroderma[Title/Abstract] OR "systemic sclerosis"[Title/Abstract] OR “diffuse cutaneous systemic sclerosis”[Title/Abstract] OR “limited cutaneous systemic sclerosis”[Title/Abstract] OR dcSSc[Title/Abstract] OR lcSSc[Title/Abstract]) **AND** (Nintedanib[Title/Abstract] OR tocilizumab[Title/Abstract] OR rituximab[Title/Abstract] OR Rituximab[MeSH Terms] OR "intravenous immunoglobulin"[Title/Abstract] OR IVIG[Title/Abstract] OR Imatinib[Title/Abstract] OR STI-571[Title/Abstract] OR STI571[Title/Abstract] OR

Abatacept[Title/Abstract] OR Romilkimab[Title/Abstract] OR SAR156597[Title/Abstract] OR Belimumab[Title/Abstract] OR fresolimumab[Title/Abstract] OR GC1008[Title/Abstract] OR rilonacept[Title/Abstract] OR Alemtuzumab[Title/Abstract] OR etanercept[Title/Abstract] OR infliximab*[Title/Abstract] OR infliximab[MeSH Terms] OR "Janus kinase inhibitor*"[Title/Abstract] OR "Janus Kinase Inhibitors"[MeSH Terms] OR ruxolitinib[Title/Abstract] OR INCB018424[Title/Abstract] OR INC424[Title/Abstract] OR tofacitinib[Title/Abstract] OR CP-690550[Title/Abstract] OR baricitinib[Title/Abstract] OR LY3009104[Title/Abstract] OR Itacitinib[Title/Abstract] OR INCB039110[Title/Abstract] OR MEDI-545[Title/Abstract] OR basiliximab[Title/Abstract] OR basiliximab[MeSH Terms] OR eculizumab[Title/Abstract] OR dasatinib[Title/Abstract] OR BMS-354825[Title/Abstract] OR Abituzumab[Title/Abstract] OR DI17E6[Title/Abstract] OR "EMD 525797"[Title/Abstract] OR AVID200[Title/Abstract] OR CM-101[Title/Abstract] OR Metelimumab[Title/Abstract] OR CAT-192[Title/Abstract] OR anakinra[Title/Abstract] OR canakinumab[Title/Abstract] OR bermekimab[Title/Abstract] OR GSK2330811[Title/Abstract] OR Nilotinib[Title/Abstract] OR AMN107[Title/Abstract] OR SAR156597[Title/Abstract] OR MEDI-551[Title/Abstract] OR inebilizumab[Title/Abstract] OR MEDI-546[Title/Abstract] OR Anifrolumab[Title/Abstract] OR FT011[Title/Abstract] OR "Brentuximab vedotin"[Title/Abstract] OR Bortezomib[Title/Abstract]) **AND** (("randomized controlled trial"[Publication Type]) OR (clinical*[Title/Abstract] AND trial*[Title/Abstract]) OR "controlled clinical trial"[Publication Type] OR "Clinical Trial"[Publication Type]) **NOT** (Review[Publication Type] OR meta-Analysis[Publication Type] OR Comment[Publication Type]) AND (English[Language])

Table S3. Final query for search in ClinicalTrial.gov database:

Filter	Search Terms
Condition/disease	Scleroderma OR "systemic sclerosis" OR "diffuse cutaneous systemic sclerosis" OR "limited cutaneous systemic sclerosis" OR dcSSc OR lcSSc
Other terms	Placebo OR "standard of care" OR cyclophosphamide
Study Results	With results

.

Table S4. Summary statistics of the covariates. Filter: count > 10

Covariate	N of trials	Mean	SD	Minimum value	Maximum value	Missingness (N)	Missingness (%)
SSc type	32	-	-	-	-	0	0
dcSSc	16	-	-	-	-	-	-
SSc	11	-	-	-	-	-	-
SSc-ILD	4	-	-	-	-	-	-
SSc-PAH	1	-	-	-	-	-	-
Age, y	31	50.5	4.6	35.6	58.2	1	3.1
Female, %	31	75.5	11.2	43.5	91.3	1	3.1
Disease duration, y	28	3.1	2.4	0.6	10.1	5	15.6

mRSS	27	22.2	5.8	9.9	34.0	5	15.6
FVC %pr	21	83.4	9.5	60.3	98.9	11	34.4
DLCO %pr	18	70.6	12.5	45.2	87.2	14	43.8
White, %	17	73.6	27.2	0.0	100.0	15	46.9
Anti-topoisomerase positive, %	16	41.4	23.4	6.0	100.0	16	50.0
HAQ-DI	12	0.9	0.3	0.3	1.4	21	62.5

Table S5: Summary of selected trials.

Author_Year	National Clinical Trial number	Clinical Phase	Interventions					Baseline (mean)						
			Drug	Dose	Frequency	RoA	MaxDur (months)	Enrollment	SSc type	Age (y)	Female (%)	DisDur (y)	mRSS (score)	FVC (%pr)
Chung_2020 [1]	NCT02161406	II	Abatacept	125 mg	QW	SC	18	88	dcSSc	51.0	75.0	1.6	22.5	85.3
Chakravarty_2015 [2]	NCT00442611	I/II	Abatacept	500-1000 mg	Q2W*3 + Q4W*4	IV	6	10	dcSSc	44.2	85.7	5.6	27.2	75.3
Khanna_2021 [3]*	NCT02745145	II	Abituzumab	500 mg; 1500 mg	Q4W	IV	12	24	SSc-ILD	55.3	79.3	3.7	-	-
Gordon_2018 [4]	NCT01670565	II	Belimumab	10 mg/kg	Q2W*3 + Q4W*10	IV	12	20	dcSSc	54.9	75.0	0.9	27.1	92.2
Solomonidi_2023 [5]	NCT04045743	II	Bermekimab	7.5 mg/kg	QW	SC	3	20	SSc	50.9	90.0	0.9	23.6	76.2
Denton_2007 [6]	NCT00043706	I/II	Metelimumab	35; 350; 700 mg	Q6W	SC	6	43	dcSSc	48.4	76.4	0.6	21.7	90.6
Denton_2022 [7]	NCT03041025	II	GSK2330811	300 mg	Q2W	SC	3	35	dcSSc	56.7	62.3	1.6	24.3	98.9
Pope_2011 [8]	NCT01545427	II	Imatinib	200 mg	BID	PO	6	10	dcSSc	49.0	83.3	4.0	34	-
Prey_2012 [9]	NCT00479934	II	Imatinib	400 mg	BID	PO	6	28	SSc	49.0	60.5	3.0	26.5	-
Schiopu_2016 [10]	NCT00946699	I	Inebilizumab	0.1; 0.3; 1; 3; 10 mg/kg	SD	IV	12	28	SSc	47.7	57.3	5.4	24.1	83.1
Takehara_2013 [11]	NCT00348296	III	Immunoglobulin	400 mg/kg/day	QD	IV	3	62	dcSSc	54.0	77.4	5.9	28.5	-
Distler_2019 [12]	NCT02597933	III	Nintedanib	150 mg	BID	PO	12	576	SSc-ILD	54.0	75.2	3.6	11.1	72.6
Mantero_2018 [13]	NCT01538719	I/II	Rilonacept	160 mg	QD*1+QW*5	SC	3	19	dcSSc	50.6	43.5	1.7	29.8	-
Boonstra_2017 [14]	-	II	Rituximab	1000 mg	0, 14, 180	IV	24	16	SSc	40.5	87.5	2.5	15.2	95.0
Ebata_2022 [15]	NCT04274257	II	Rituximab	375 mg/m2	Q2W	IV	6	54	SSc	48.7	90.8	10.1	15.1	88.7
Zamanian_2021 [16]*	NCT01086540	II	Rituximab	1000 mg	Q2W	IV	12	57	SSc-PAH	58.2	91.3	-	-	-
Sircar_2018 [17]	-	III	Rituximab	1000 mg	Q2W	IV	6	60	dcSSc	35.6	83.0	1.9	22.8	60.3
Daoussis_2010 [18]	-	-	Rituximab	375 mg/m2	QW	IV	6	14	SSc-ILD	55.5	-	7.6	12.5	77.1
Allanore_2020 [19]	NCT02921971	II	Romilkimab	200 mg	QW	IV	6	97	dcSSc	49.8	79.5	1.7	20.6	92.8
Khanna_2022 [20]	NCT02453256	III	Tocilizumab	162 mg	QW	SC	12	210	SSc	50.6	73.2	2.0	22.0	84.7
Khanna_2018 [21]	NCT01532869	II	Tocilizumab	162 mg	QW	SC	12	87	SSc	49.5	77.0	1.5	26.0	81.0
Shima_2019 [22]	-	II	Tocilizumab	8 mg/kg	Q4W	IV	6	13	SSc	48.2	78.6	7.0	25.6	76.3
Khanna_2022 [23]	NCT03274076	I/II	Tofacitinib	5 mg	BID	PO	6	15	SSc	50.6	73.2	2.0	22.0	84.7
Karalilova_2021 [24]	-	II	Tofacitinib	5 mg	BID	PO	12	66	SSc	48.3	89.5	2.9	23.5	-
-	NCT04647890 [25]	II	Asengeprast	200; 400 mg	QD	PO	3	30	dcSSc	-	73.3	-	-	-
-	NCT02370693 [26]	II	Bortezomib	1.3 mg/m2	QD*2 + Q4D*6	IV	6	7	SSc	55.8	58.4	-	-	-
Chen_2025 [27]	-	II	Baricitinib	2; 4 mg	QD	PO	3	48	dcSSc	52.2	79.1	-	17.8	83.2
-	NCT04683029 [28]	II	Guselkumab	400 mg, then 200 mg	Q4W	SC	6	56	dcSSc	51.0	83.8	-	-	-
Distler_2023 [29]	NCT02283762	IIb	Riociguat	0.5 - 2.5 mg	TID	PO	12	121	dcSSc	50.7	76.0	0.8	16.8	92.8
Khanna_2023 [30]	NCT03798366	IIa	Ziritaxestat	600 mg	QD	PO	6	33	dcSSc	48.8	69.1	2.0	24.8	90.8
Allanore_2018 [31]	NCT01651143	II	Fipaxalparant	300 mg	BID	PO	2	32	dcSSc	49.7	65.3	1.7	23.8	-
-	NCT03221257 [32]	II	Pirfenidone	801 mg/day	TID	PO	18	51	SSc-ILD	54.6	70.6	2.6	9.9	71.1

Abbreviations: RoA = route of administration, MaxDur = maximum duration, y = years, DisDur = disease duration, %pr = % predicted, QW = once weekly, Q2W = once every 2 weeks, Q4W = once every 4 weeks, Q6W = every 6 weeks, QD = daily, BID = twice daily, TID = three times a day, SD = single dose, Q2W3 + Q4W4 = three doses every 2 weeks then four doses every 4 weeks, Q2W3 +

Q4W10 = three doses every 2 weeks then ten doses every 4 weeks, QD1 + QW5 = one daily dose then five weekly doses, QD2 + Q4D6 = two daily doses then six doses every 4 days, 0, 14, 180 = doses on days 0, 14, 180, SC = subcutaneous, IV = intravenous, PO = oral. dcSSc - diffuse subcutaneous SSc, SSc-ILD - SSc-associated ILD, SSc-PAH - SSc-associated pulmonary arterial hypertension. SSc indicates that a study population may include patients with various SSc forms. * indicates studies that were not included in the meta-analysis due to the absence of mRSS or FVC data and appeared only in the exploratory analysis.

Table S6. Drug targets.

Target	Drugs	Total	Reference
Interleukins	Bermekimab (IL-1α), Rilonacept (IL-1), Tocilizumab (IL-6 receptor), Romilkimab (IL-4/IL-13), Guselkumab (IL-23)	5	[33,34]
B-cell Survival	Belimumab (BAFF/BLyS), Rituximab (CD20), Inebilizumab (CD19)	3	[34]
T-cell co-stimulation	Abatacept (CD80/CD86)	1	[34]
JAK/STAT Signaling	Tofacitinib (JAK), Baricitinib (JAK1/2)	2	[34,35]
TGF-β/Fibrosis	Metelimumab (TGF-β1), GSK2330811 (Oncostatin M), Asengeprast (GPR68), Abituzumab (CD51), Ziritaxestat (autotaxin), Riociguat (cGC), Fipaxalparant (LPAR1), Pirfenidone (TGF-β)	8	[36–41]
Tyrosine Kinase Signaling	Nintedanib (PDGFR, FGFR, VEGFR), Imatinib (CSF1R, ABL, c-KIT, FLT3, PDGFR-β)	2	[34,42]
Proteostasis	Bortezomib (proteasome proteins)	1	[43]
Autoantibodies and Fcγ receptor pathways	Immunoglobulin (immunomodulation)	1	[44]

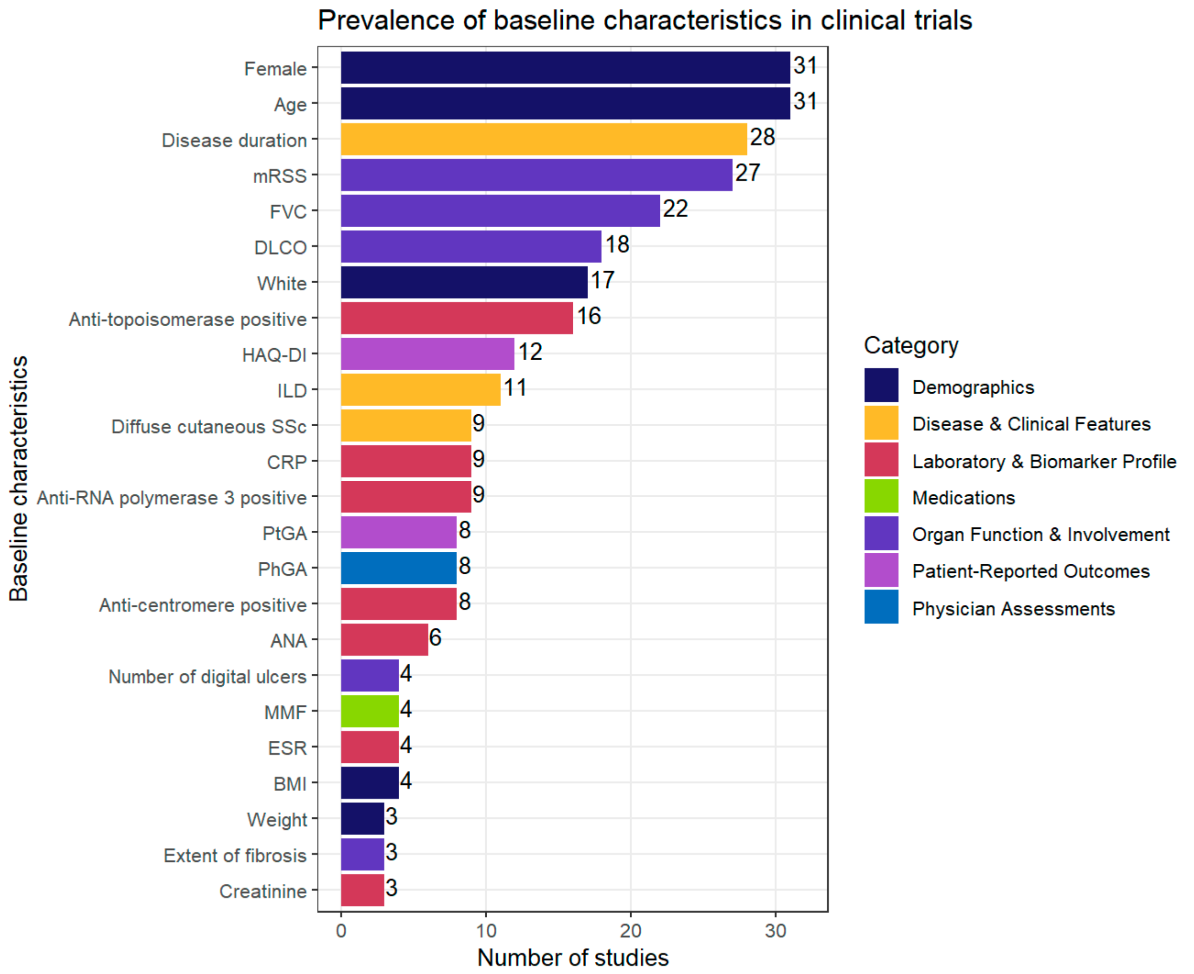

Figure S1. Prevalence of baseline characteristics in clinical trials filtered to include only those reported in more than two studies (count > 2). Abbreviations: mRSS, modified Rodnan skin score; FVC, forced vital capacity; DLCO, diffusing capacity of the lungs for carbon monoxide; HAQ-DI, Health

Assessment Questionnaire-Disability Index; ILD, interstitial lung disease; SSc, systemic sclerosis; CRP, C-reactive protein; PtGA, Patient Global Assessment; PhGA, Physician Global Assessment; ANA, antinuclear antibody; MMF, mycophenolate mofetil; ESR, erythrocyte sedimentation rate; BMI, body mass index.

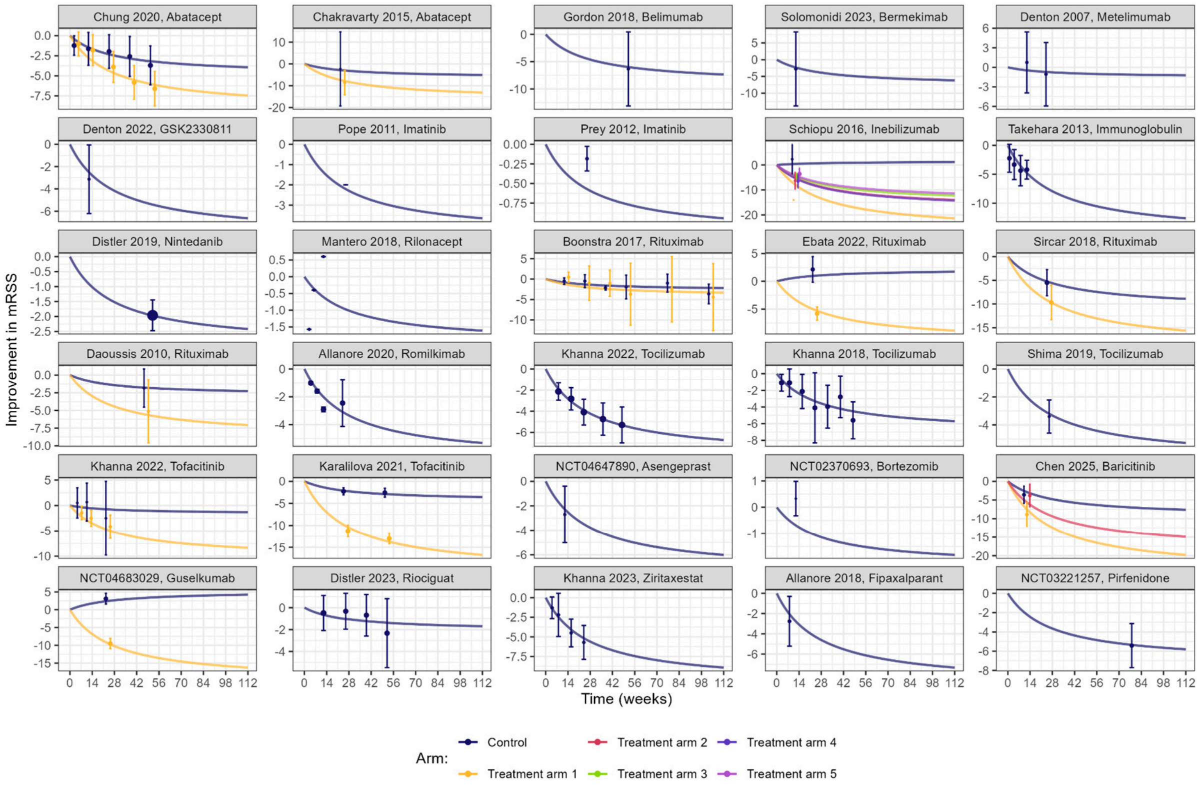

Figure S2. Individual fits for SSc endpoint (mRSS) per studies with observed data points sized by the number of patients in arm. Abbreviations: mRSS, modified Rodan skin score.

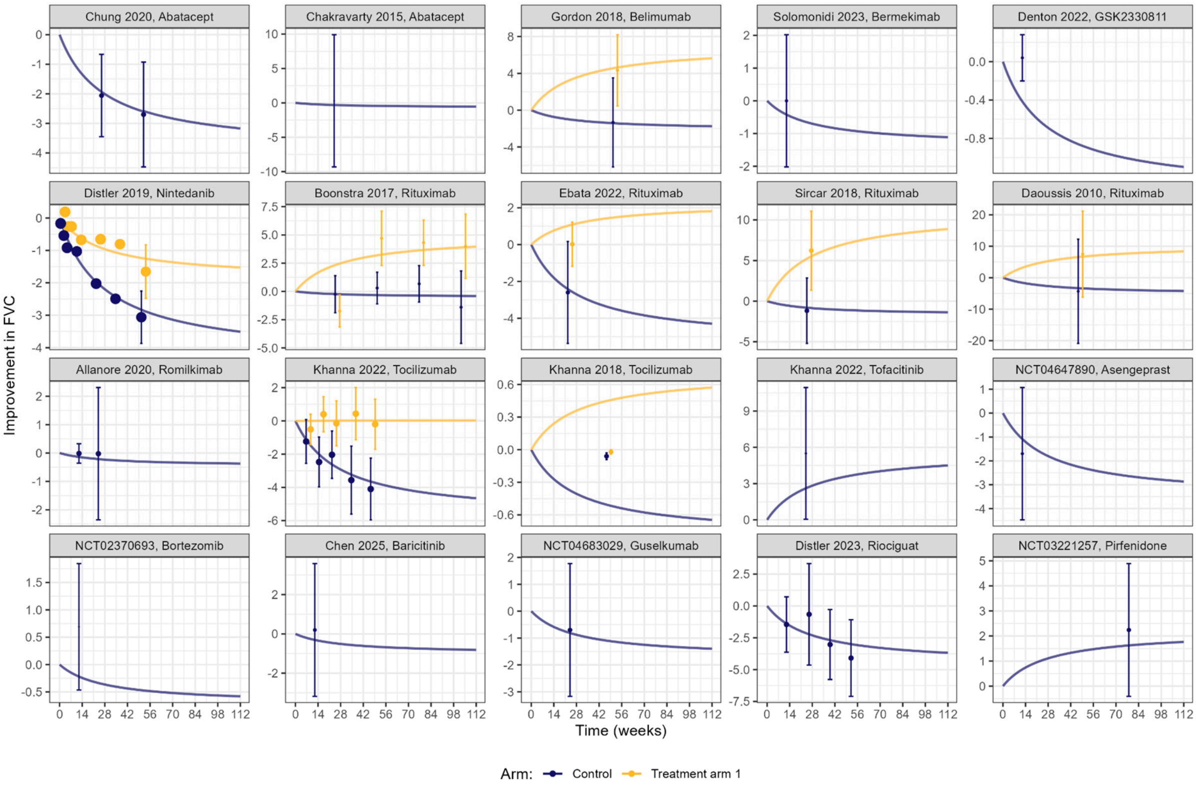

Figure S3. Individual fits for SSc endpoint (FVC) per studies with observed data points sized by the number of patients in arm. Abbreviations: FVC, forced vital capacity

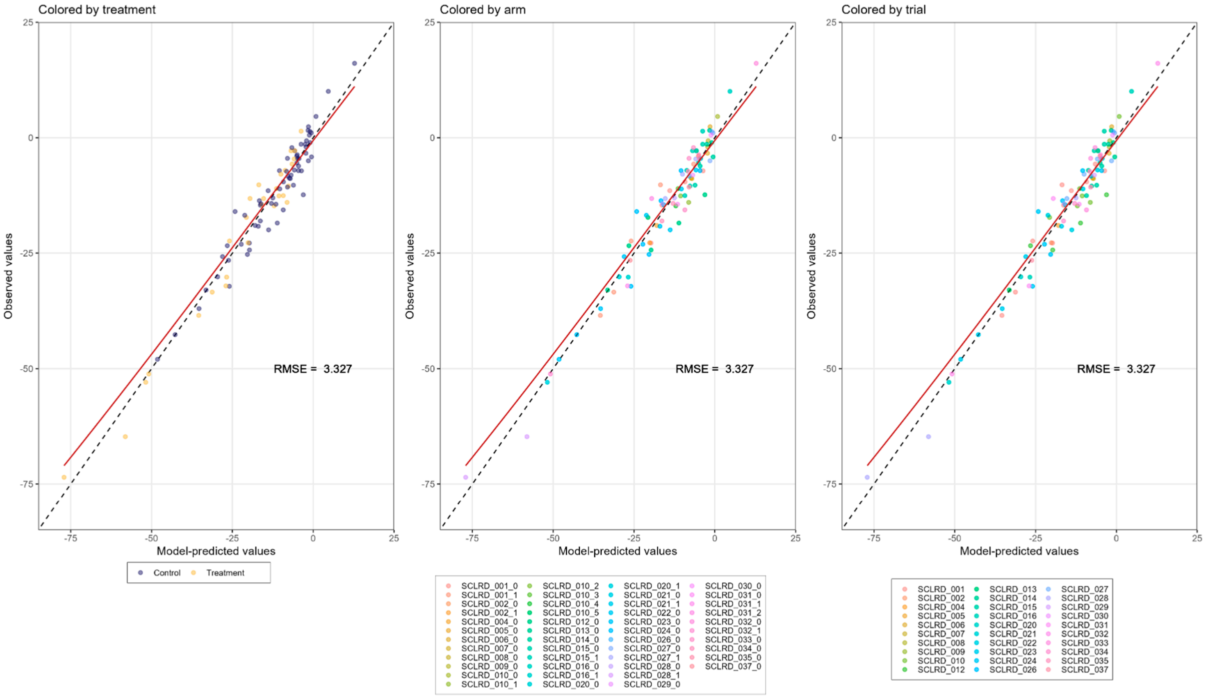

Figure S4. Observed data versus predicted values for mRSS score. Abbreviations: mRSS, modified Rodan skin score.

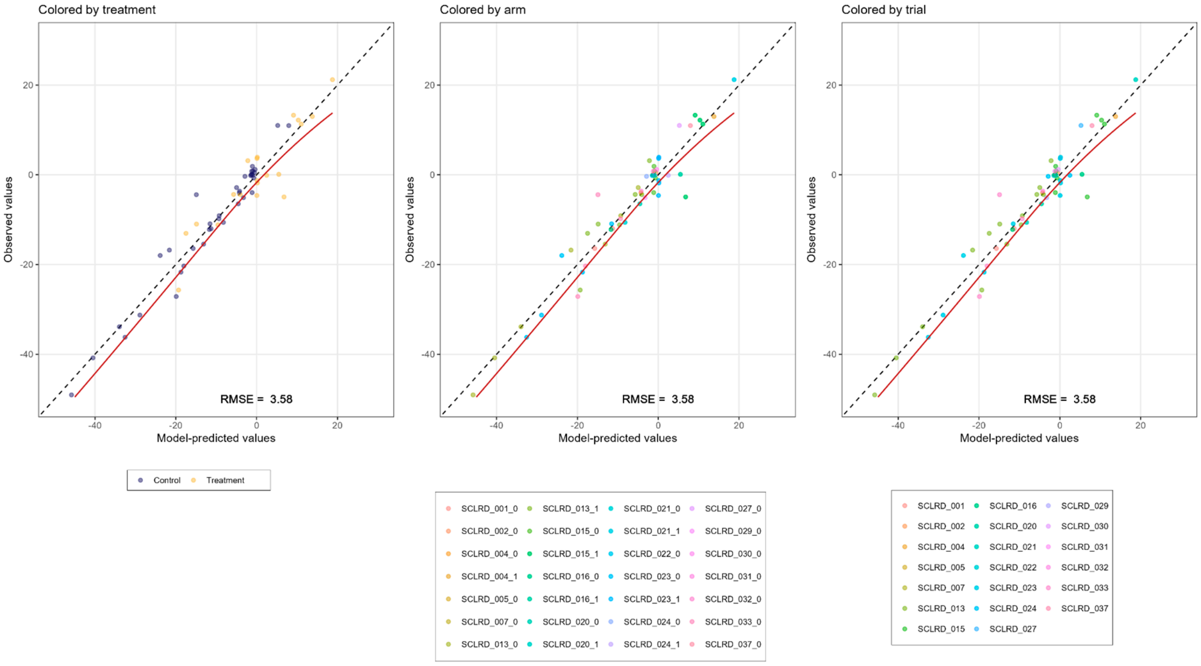

Figure S5. Observed data versus predicted values for FVC score. Abbreviations: FVC, forced vital capacity

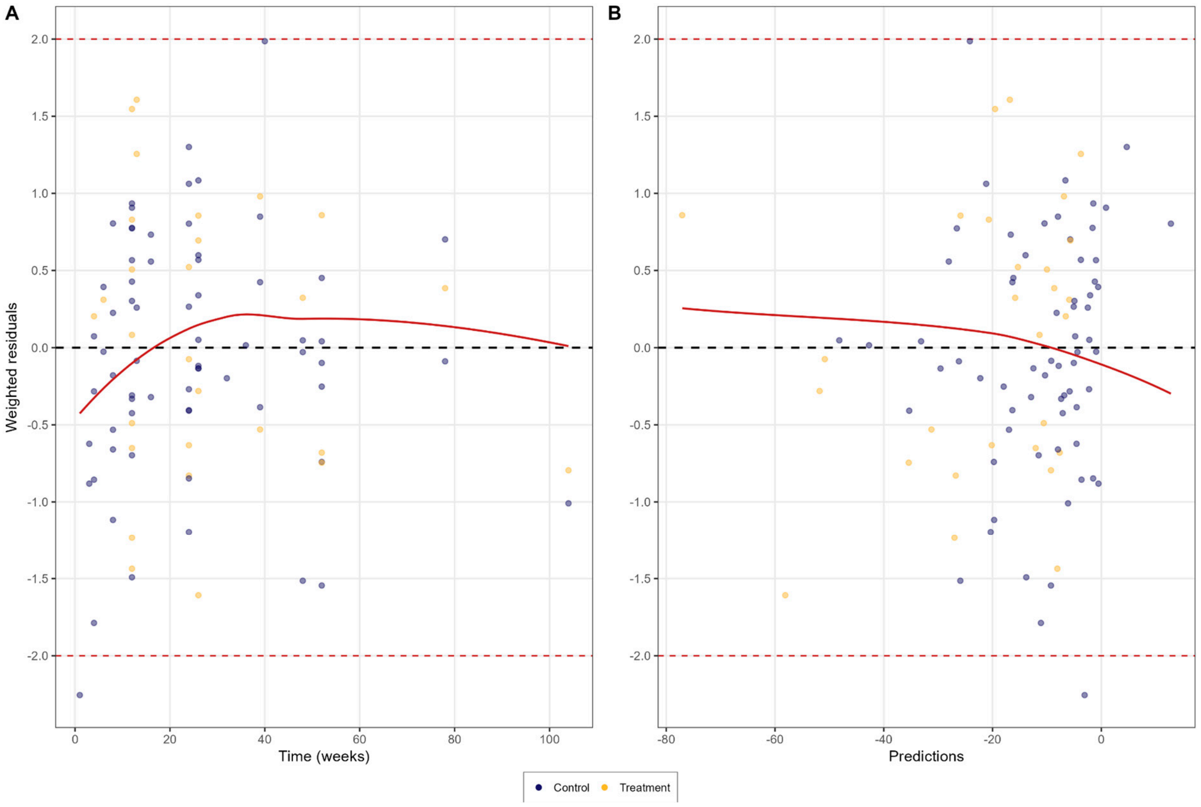

Figure S6. Weighted residuals distribution from time (A) and predicted values (B) for mRSS score. Abbreviations: mRSS, modified Rodan skin score.

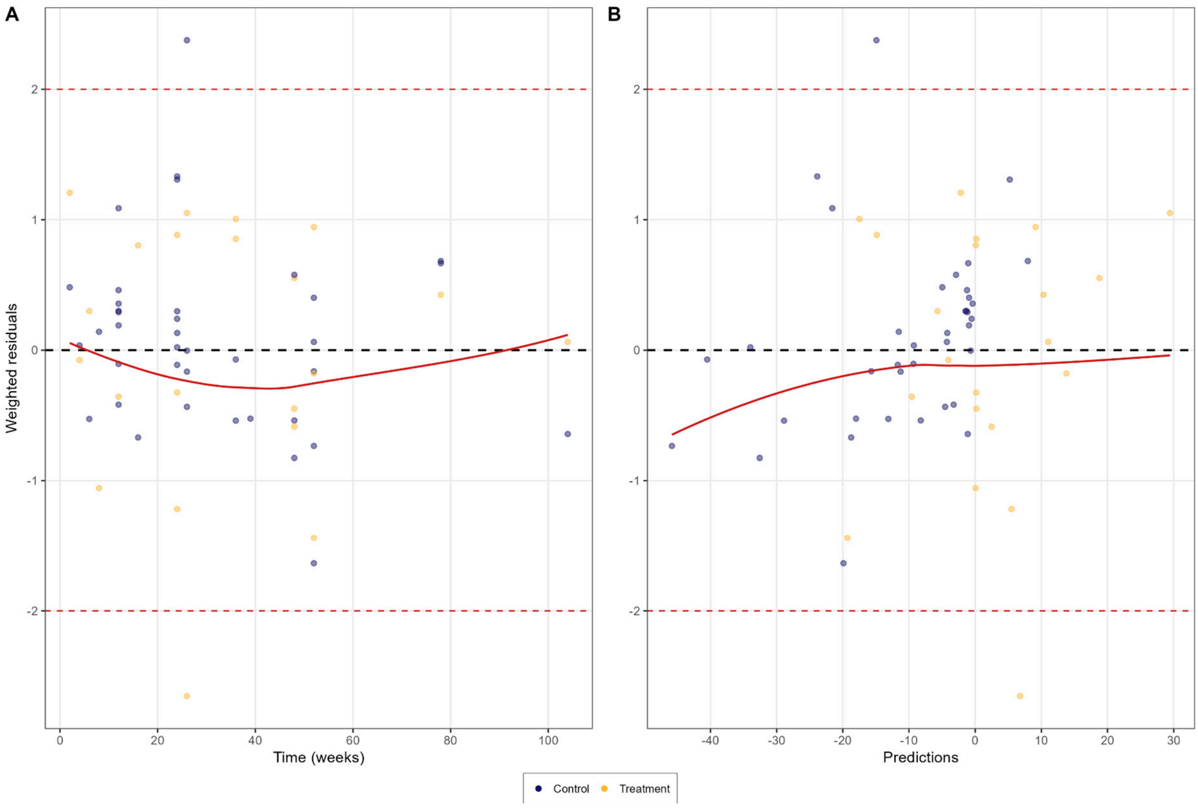

Figure S7. Weighted residuals distribution from time (A) and predicted values (B) for FVC score. Abbreviations: FVC, forced vital capacity

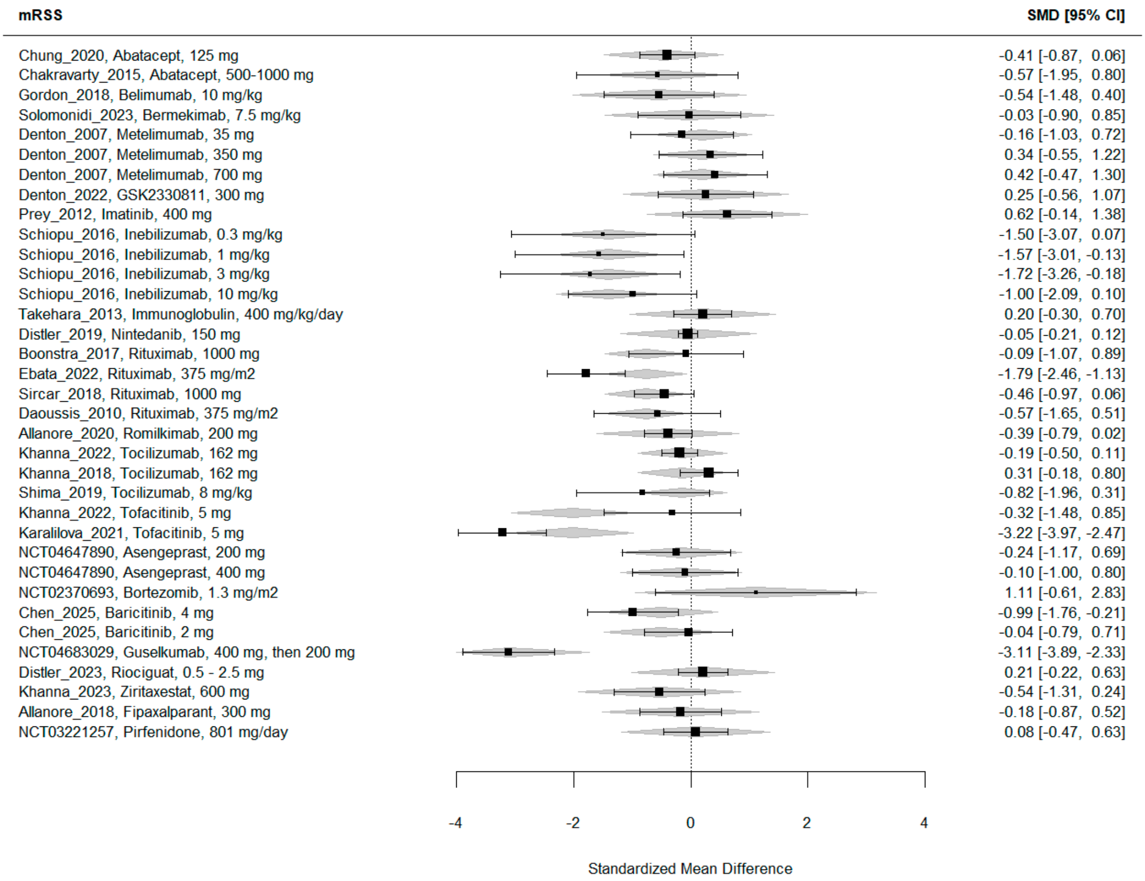

Figure S8. Meta-analysis results for mRSS using final study timepoint and metafor package in R. Abbreviations: mRSS, modified Rodan skin score.

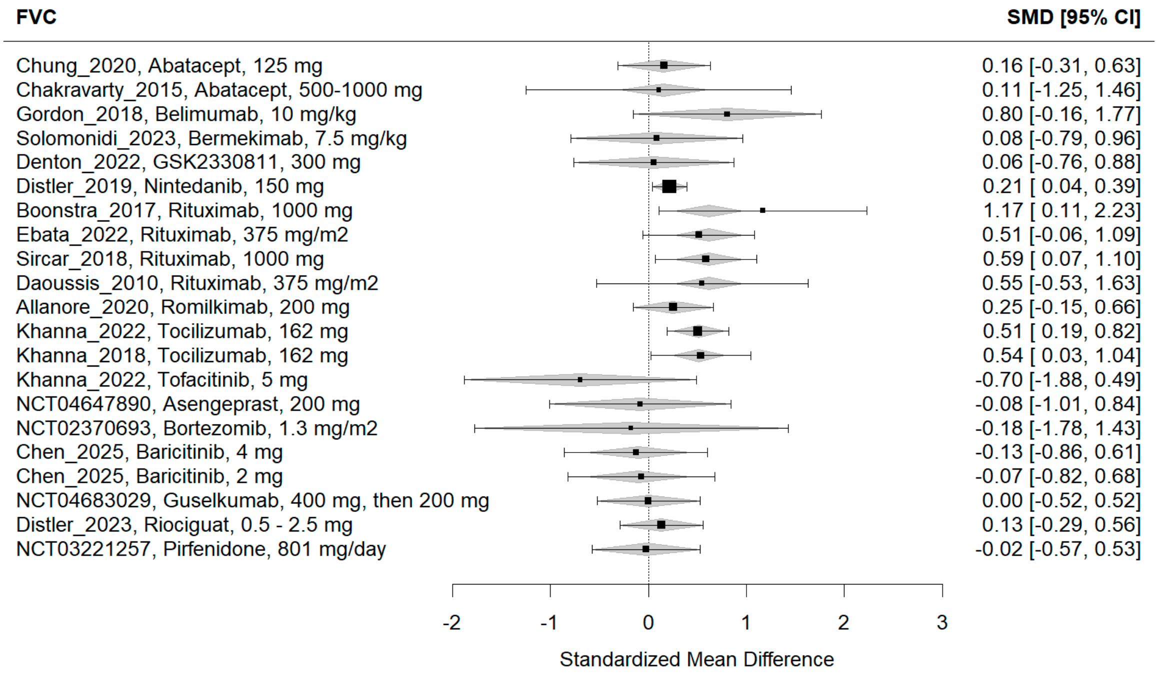

Figure S9. Meta-analysis results for FVC using final study timepoint and metafor package in R. Abbreviations: FVC, forced vital capacity

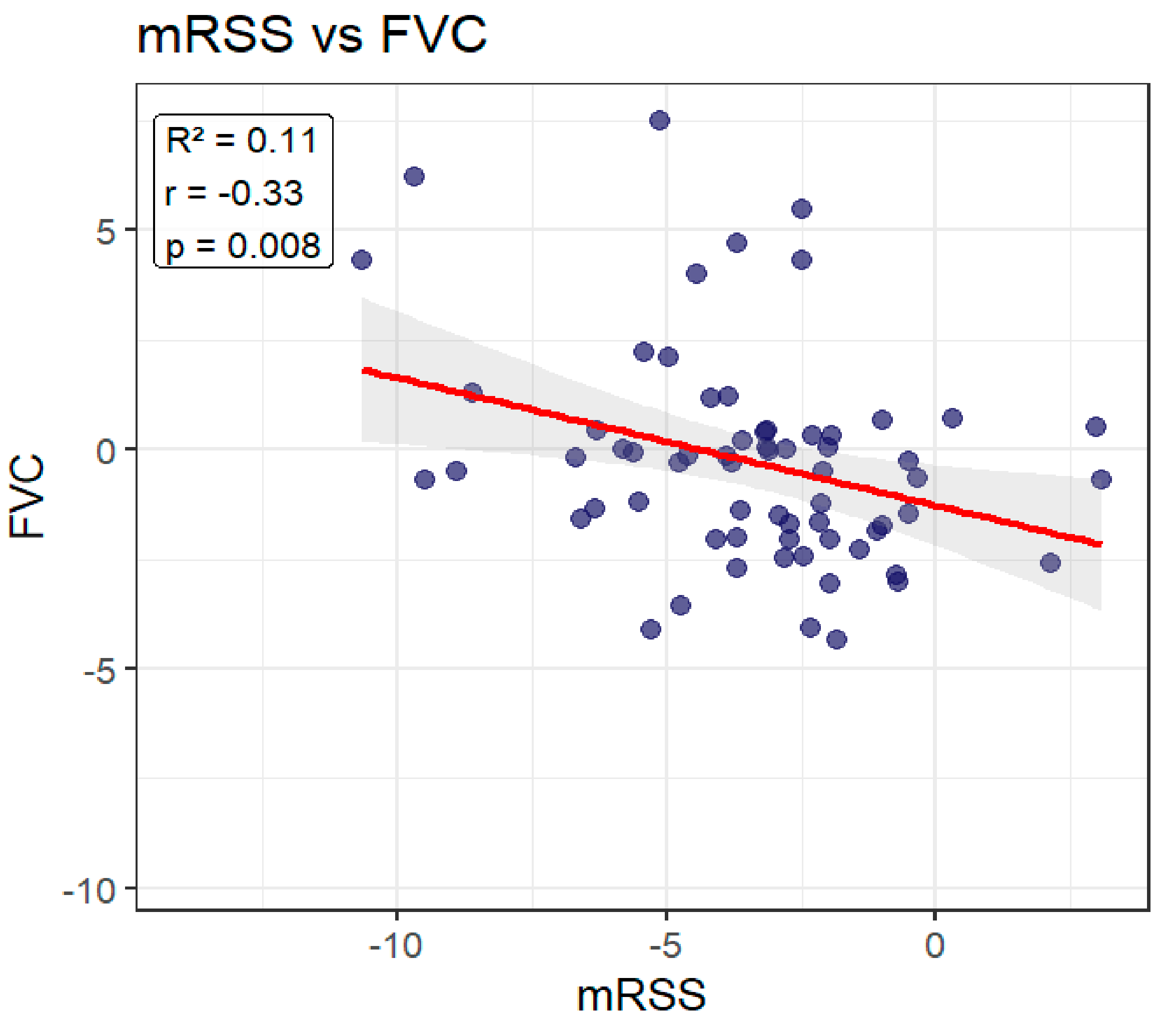

Figure S10. Correlation between changes in modified Rodnan skin score (mRSS) and forced vital capacity (FVC) across pooled trial arms. Each point represents an arm-level paired mean change from baseline in mRSS and FVC, with the red line indicating the fitted linear regression and the shaded band its 95% confidence interval. A modest but statistically significant negative association was observed ($r = -0.33$, $R^2 = 0.11$, $p = 0.008$), suggesting that greater improvements in skin fibrosis tend to coincide with stabilisation or improvement in lung function.

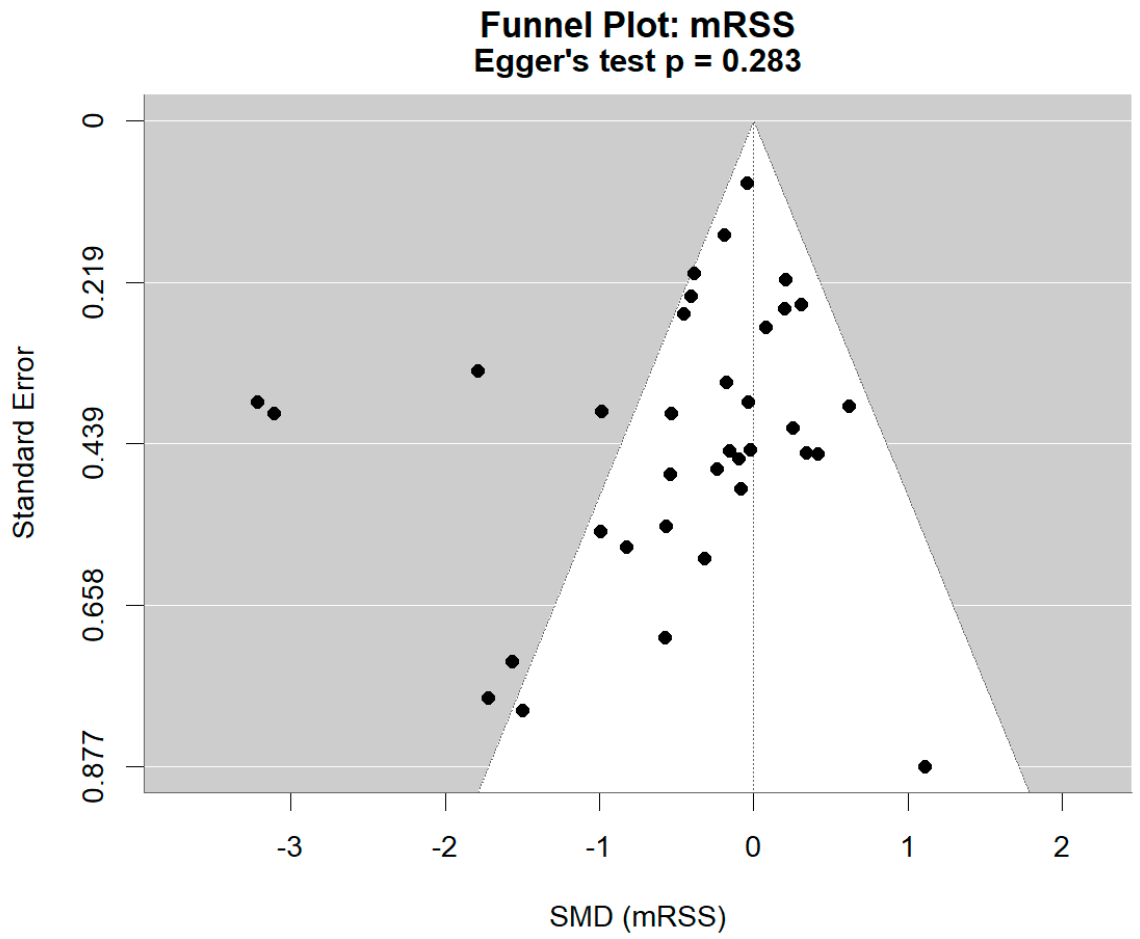

Figure S11. Funnel plot assessing publication bias in standardized mean difference (SMD) of modified Rodnan Skin Score (mRSS) for systemic sclerosis treatments. Each point represents an individual trial results at final timepoint.

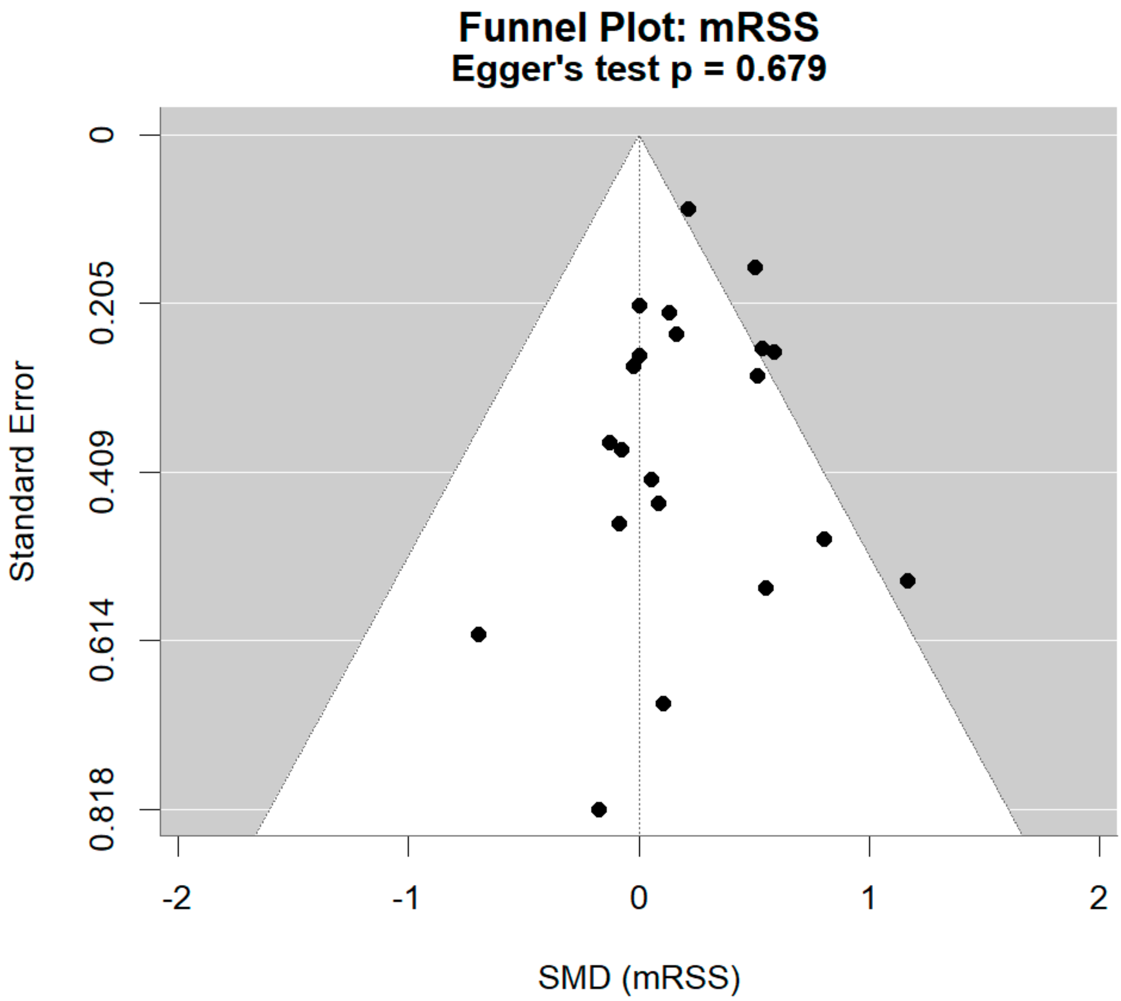

Figure S12. Funnel plot assessing publication bias in standardized mean difference (SMD) of forced vital capacity (FVC) % predicted for systemic sclerosis treatments. Individual trial estimates plotted against standard error; symmetrical distribution indicates low publication bias risk.

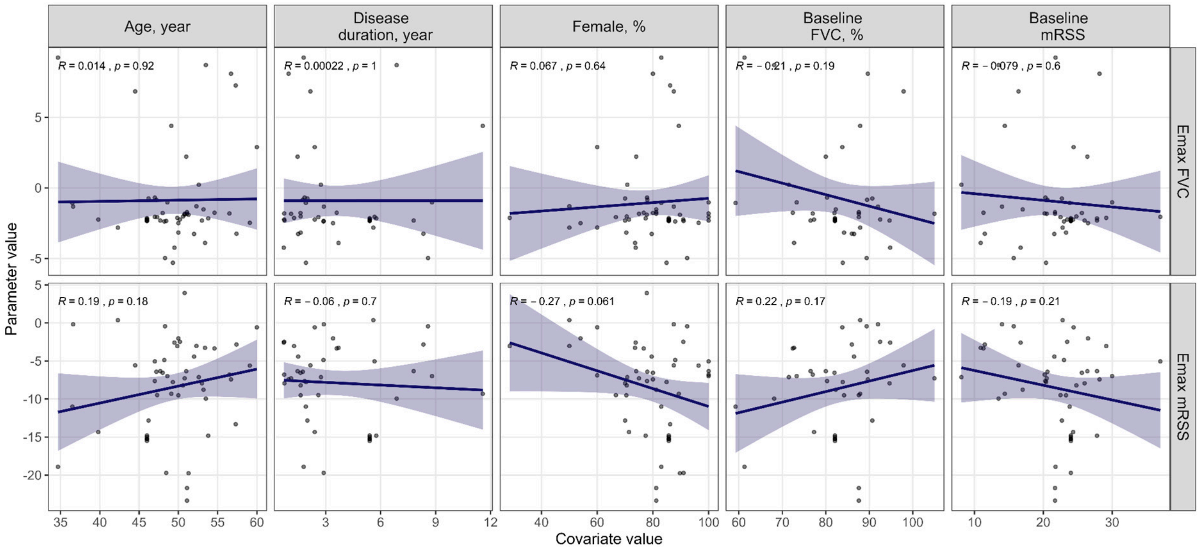

Figure S13. Correlation of individual parameter estimates with baseline covariates.

References

1. Chung, L.; Spino, C.; McLain, R.; Johnson, S.R.; Denton, C.P.; Molitor, J.A.; Steen, V.D.; Lafyatis, R.; Simms, R.W.; Kafaja, S.; et al. Safety and Efficacy of Abatacept in Early Diffuse Cutaneous Systemic Sclerosis (ASSET): Open-Label Extension of a Phase 2, Double-Blind Randomised Trial. *Lancet Rheumatol* **2020**, *2*, e743–e753, doi:10.1016/S2665-9913(20)30237-X.
2. Chakravarty, E.F.; Martyanov, V.; Fiorentino, D.; Wood, T.A.; Haddon, D.J.; Jarrell, J.A.; Utz, P.J.; Genovese, M.C.; Whitfield, M.L.; Chung, L. Gene Expression Changes Reflect Clinical Response in a Placebo-Controlled Randomized Trial of Abatacept in Patients with Diffuse Cutaneous Systemic Sclerosis. *Arthritis Res Ther* **2015**, *17*, 159, doi:10.1186/s13075-015-0669-3.
3. Khanna, D.; Tashkin, D.P.; Wells, A.U.; Seibold, J.R.; Wax, S.; Vazquez-Mateo, C.; Fleuranceau-Morel, P.; Damian, D.; Denton, C.P. STRATUS: A Phase II Study of Abituzumab in Patients With Systemic Sclerosis-Associated Interstitial Lung Disease. *J Rheumatol* **2021**, *48*, 1295–1298, doi:10.3899/jrheum.191365.
4. Gordon, J.K.; Martyanov, V.; Franks, J.M.; Bernstein, E.J.; Szymonifka, J.; Magro, C.; Wildman, H.F.; Wood, T.A.; Whitfield, M.L.; Spiera, R.F. Belimumab for the Treatment of Early Diffuse Systemic Sclerosis: Results of a Randomized, Double-Blind, Placebo-Controlled, Pilot Trial. *Arthritis Rheumatol* **2018**, *70*, 308–316, doi:10.1002/art.40358.
5. Solomonidi, N.; Vlachoyiannopoulos, P.G.; Pappa, M.; Liantinioti, G.; Ktena, S.; Theotikos, E.; Elezoglou, A.; Netea, M.G.; Giamarellos-Bourboulis, E.J. A Randomized Clinical Trial of Bermekimab Treatment for Clinical Improvement of Systemic Sclerosis. *iScience* **2023**, *26*, 107670, doi:10.1016/j.isci.2023.107670.
6. Denton, C.P.; Merkel, P.A.; Furst, D.E.; Khanna, D.; Emery, P.; Hsu, V.M.; Silliman, N.; Streisand, J.; Powell, J.; Akesson, A.; et al. Recombinant Human Anti-Transforming Growth Factor Beta1 Antibody Therapy in Systemic Sclerosis: A Multicenter, Randomized, Placebo-Controlled Phase I/II Trial of CAT-192. *Arthritis Rheum* **2007**, *56*, 323–333, doi:10.1002/art.22289.
7. Denton, C.P.; Del Galdo, F.; Khanna, D.; Vonk, M.C.; Chung, L.; Johnson, S.R.; Varga, J.; Furst, D.E.; Temple, J.; Zecchin, C.; et al. Biological and Clinical Insights from a Randomized Phase 2 Study of an Anti-Oncostatin M Monoclonal Antibody in Systemic Sclerosis. *Rheumatology (Oxford)* **2022**, *62*, 234–242, doi:10.1093/rheumatology/keac300.
8. Pope, J.; McBain, D.; Petrlich, L.; Watson, S.; Vanderhoek, L.; de Leon, F.; Seney, S.; Summers, K. Imatinib in Active Diffuse Cutaneous Systemic Sclerosis: Results of a Six-Month, Randomized, Double-Blind, Placebo-Controlled, Proof-of-Concept Pilot Study at a Single Center. *Arthritis Rheum* **2011**, *63*, 3547–3551, doi:10.1002/art.30549.
9. Prey, S.; Ezzedine, K.; Doussau, A.; Grandoulier, A.-S.; Barcat, D.; Chatelus, E.; Diot, E.; Durant, C.; Hachulla, E.; de Korwin-Krokowski, J.-D.; et al. Imatinib Mesylate in Scleroderma-Associated Diffuse Skin Fibrosis: A Phase II Multicentre Randomized Double-Blinded Controlled Trial. *Br J Dermatol* **2012**, *167*, 1138–1144, doi:10.1111/j.1365-2133.2012.11186.x.
10. Schiopu, E.; Chatterjee, S.; Hsu, V.; Flor, A.; Cimbora, D.; Patra, K.; Yao, W.; Li, J.; Streicher, K.; McKeever, K.; et al. Safety and Tolerability of an Anti-CD19 Monoclonal Antibody, MEDI-551, in Subjects with Systemic Sclerosis: A Phase I, Randomized, Placebo-Controlled, Escalating Single-Dose Study. *Arthritis Res Ther* **2016**, *18*, 131, doi:10.1186/s13075-016-1021-2.
11. Takehara, K.; Ihn, H.; Sato, S. A Randomized, Double-Blind, Placebo-Controlled Trial: Intravenous Immunoglobulin Treatment in Patients with Diffuse Cutaneous Systemic Sclerosis. *Clin Exp Rheumatol* **2013**, *31*, 151–156.
12. Distler, O.; Highland, K.B.; Gahlemann, M.; Azuma, A.; Fischer, A.; Mayes, M.D.; Raghu, G.; Sauter, W.; Girard, M.; Alves, M.; et al. Nintedanib for Systemic Sclerosis-Associated Interstitial Lung Disease. *N Engl J Med* **2019**, *380*, 2518–2528, doi:10.1056/NEJMoa1903076.
13. Mantero, J.C.; Kishore, N.; Ziemek, J.; Stifano, G.; Zammitti, C.; Khanna, D.; Gordon, J.K.; Spiera, R.; Zhang, Y.; Simms, R.W.; et al. Randomised, Double-Blind, Placebo-Controlled Trial of IL1-Trap, Rilonacept, in Systemic Sclerosis. A Phase I/II Biomarker Trial. *Clin Exp Rheumatol* **2018**, *36 Suppl 113*, 146–149.
14. Boonstra, M.; Meijs, J.; Dorjée, A.L.; Marsan, N.A.; Schouffoer, A.; Ninaber, M.K.; Quint, K.D.; Bonte-Mineur, F.; Huizinga, T.W.J.; Scherer, H.U.; et al. Rituximab in Early Systemic Sclerosis. *RMD Open* **2017**, *3*, e000384, doi:10.1136/rmdopen-2016-000384.
15. Ebata, S.; Yoshizaki, A.; Oba, K.; Kashiwabara, K.; Ueda, K.; Uemura, Y.; Watadani, T.; Fukasawa, T.; Miura, S.; Yoshizaki-Ogawa, A.; et al. Safety and Efficacy of Rituximab in Systemic Sclerosis (DESIRES): Open-Label Extension of a Double-Blind, Investigators-Initiated, Randomised, Placebo-Controlled Trial. *Lancet Rheumatol* **2022**, *4*, e546–e555, doi:10.1016/S2665-9913(22)00131-X.

16. Zamanian, R.T.; Badesch, D.; Chung, L.; Domsic, R.T.; Medsger, T.; Pinckney, A.; Keyes-Elstein, L.; D'Aveta, C.; Spychala, M.; White, R.J.; et al. Safety and Efficacy of B-Cell Depletion with Rituximab for the Treatment of Systemic Sclerosis-Associated Pulmonary Arterial Hypertension: A Multicenter, Double-Blind, Randomized, Placebo-Controlled Trial. *Am J Respir Crit Care Med* **2021**, *204*, 209–221, doi:10.1164/rccm.202009-3481OC.
17. Sircar, G.; Goswami, R.P.; Sircar, D.; Ghosh, A.; Ghosh, P. Intravenous Cyclophosphamide vs Rituximab for the Treatment of Early Diffuse Scleroderma Lung Disease: Open Label, Randomized, Controlled Trial. *Rheumatology (Oxford)* **2018**, *57*, 2106–2113, doi:10.1093/rheumatology/key213.
18. Daoussis, D.; Liossis, S.-N.C.; Tsamandas, A.C.; Kalogeropoulou, C.; Kazantzi, A.; Sirinian, C.; Karampetsou, M.; Yiannopoulos, G.; Andonopoulos, A.P. Experience with Rituximab in Scleroderma: Results from a 1-Year, Proof-of-Principle Study. *Rheumatology (Oxford)* **2010**, *49*, 271–280, doi:10.1093/rheumatology/kep093.
19. Allanore, Y.; Wung, P.; Soubrane, C.; Esperet, C.; Marrache, F.; Bejuit, R.; Lahmar, A.; Khanna, D.; Denton, C.P.; Investigators A Randomised, Double-Blind, Placebo-Controlled, 24-Week, Phase II, Proof-of-Concept Study of Romilkimab (SAR156597) in Early Diffuse Cutaneous Systemic Sclerosis. *Ann Rheum Dis* **2020**, *79*, 1600–1607, doi:10.1136/annrheumdis-2020-218447.
20. Khanna, D.; Lin, C.J.F.; Furst, D.E.; Wagner, B.; Zucchetto, M.; Raghu, G.; Martinez, F.J.; Goldin, J.; Siegel, J.; Denton, C.P. Long-Term Safety and Efficacy of Tocilizumab in Early Systemic Sclerosis-Interstitial Lung Disease: Open-Label Extension of a Phase 3 Randomized Controlled Trial. *Am J Respir Crit Care Med* **2022**, *205*, 674–684, doi:10.1164/rccm.202103-0714OC.
21. Khanna, D.; Denton, C.P.; Lin, C.J.F.; van Laar, J.M.; Frech, T.M.; Anderson, M.E.; Baron, M.; Chung, L.; Fierlbeck, G.; Lakshminarayanan, S.; et al. Safety and Efficacy of Subcutaneous Tocilizumab in Systemic Sclerosis: Results from the Open-Label Period of a Phase II Randomised Controlled Trial (faSScinate). *Ann Rheum Dis* **2018**, *77*, 212–220, doi:10.1136/annrheumdis-2017-211682.
22. Shima, Y.; Kawaguchi, Y.; Kuwana, M. Add-on Tocilizumab versus Conventional Treatment for Systemic Sclerosis, and Cytokine Analysis to Identify an Endotype to Tocilizumab Therapy. *Mod Rheumatol* **2019**, *29*, 134–139, doi:10.1080/14397595.2018.1452178.
23. Khanna, D.; Padilla, C.; Tsoi, L.C.; Nagaraja, V.; Khanna, P.P.; Tabib, T.; Kahlenberg, J.M.; Young, A.; Huang, S.; Gudjonsson, J.E.; et al. Tofacitinib Blocks IFN-Regulated Biomarker Genes in Skin Fibroblasts and Keratinocytes in a Systemic Sclerosis Trial. *JCI Insight* **2022**, *7*, e159566, doi:10.1172/jci.insight.159566.
24. Karalilova, R.V.; Batalov, Z.A.; Sapundzhieva, T.L.; Matucci-Cerinic, M.; Batalov, A.Z. Tofacitinib in the Treatment of Skin and Musculoskeletal Involvement in Patients with Systemic Sclerosis, Evaluated by Ultrasound. *Rheumatol Int* **2021**, *41*, 1743–1753, doi:10.1007/s00296-021-04956-7.
25. Certa Therapeutics *A Phase II, Randomised, Double Blind, Placebo-Controlled Study of the Pharmacokinetics, Pharmacodynamic Effects, and Safety, of Oral FT011 in Participants With Diffuse Systemic Sclerosis*; clinicaltrials.gov, 2023;
26. Jain, M. *Comparing and Combining Bortezomib and Mycophenolate in SSc Pulmonary Fibrosis Grant Number: R34HL122558*; clinicaltrials.gov, 2021;
27. Chen, F.; Ye, W.; Wang, Q.; Zhao, L.; Liang, M.; Zheng, S.; Zhao, T.; Xuan, D.; Zhu, Z.; Yu, Y.; et al. BAricitinib in Patients with SystemIC Sclerosis (BASICS): A Prospective, Open-Label, Randomised Trial. *Clin Rheumatol* **2025**, *44*, 2861–2871, doi:10.1007/s10067-025-07433-9.
28. Janssen Pharmaceutical K.K. *A Multicenter, Randomized, Placebo-Controlled, Double-Blind, Proof-of-Concept Study of Guselkumab in Participants With Systemic Sclerosis*; clinicaltrials.gov, 2025;
29. Distler, O.; Allanore, Y.; Denton, C.P.; Kuwana, M.; Matucci-Cerinic, M.; Pope, J.E.; Atsumi, T.; Bečvář, R.; Czirják, L.; Hachulla, E.; et al. Riociguat in Patients with Early Diffuse Cutaneous Systemic Sclerosis (RISE-SSc): Open-Label, Long-Term Extension of a Phase 2b, Randomised, Placebo-Controlled Trial. *Lancet Rheumatol* **2023**, *5*, e660–e669, doi:10.1016/S2665-9913(23)00238-2.
30. Khanna, D.; Denton, C.P.; Furst, D.E.; Mayes, M.D.; Matucci-Cerinic, M.; Smith, V.; de Vries, D.; Ford, P.; Bauer, Y.; Randall, M.J.; et al. A 24-Week, Phase IIa, Randomized, Double-Blind, Placebo-Controlled Study of Ziritaxestat in Early Diffuse Cutaneous Systemic Sclerosis. *Arthritis Rheumatol* **2023**, *75*, 1434–1444, doi:10.1002/art.42477.
31. Allanore, Y.; Distler, O.; Jagerschmidt, A.; Illiano, S.; Ledein, L.; Boitier, E.; Agueusop, I.; Denton, C.P.; Khanna, D. Lysophosphatidic Acid Receptor 1 Antagonist SAR100842 for Patients With Diffuse Cutaneous Systemic Sclerosis: A Double-Blind, Randomized, Eight-Week Placebo-Controlled Study Followed by a Sixteen-Week Open-Label Extension Study. *Arthritis Rheumatol* **2018**, *70*, 1634–1643, doi:10.1002/art.40547.
32. Roth, M. *Scleroderma Lung Study III (SLS III): Combining the Anti-Fibrotic Effects of Pirfenidone (PFD) With Mycophenolate (MMF) for Treating Scleroderma-Related Interstitial Lung Disease*; clinicaltrials.gov, 2023;

33. Kurzrock, R.; Hickish, T.; Wyrwicz, L.; Saunders, M.; Wu, Q.; Stecher, M.; Mohanty, P.; Dinarello, C.A.; Simard, J. Interleukin-1 Receptor Antagonist Levels Predict Favorable Outcome after Bermekimab, a First-in-Class True Human Interleukin-1α Antibody, in a Phase III Randomized Study of Advanced Colorectal Cancer. *Oncoimmunology* **2019**, *8*, 1551651, doi:10.1080/2162402X.2018.1551651.
34. Campochiaro, C.; Allanore, Y. An Update on Targeted Therapies in Systemic Sclerosis Based on a Systematic Review from the Last 3 Years. *Arthritis Res Ther* **2021**, *23*, 155, doi:10.1186/s13075-021-02536-5.
35. Hou, Z.; Su, X.; Han, G.; Xue, R.; Chen, Y.; Chen, Y.; Wang, H.; Yang, B.; Liang, Y.; Ji, S. JAK1/2 Inhibitor Baricitinib Improves Skin Fibrosis and Digital Ulcers in Systemic Sclerosis. *Front Med (Lausanne)* **2022**, *9*, 859330, doi:10.3389/fmed.2022.859330.
36. Lord, D.M.; Bird, J.J.; Honey, D.M.; Best, A.; Park, A.; Wei, R.R.; Qiu, H. Structure-Based Engineering to Restore High Affinity Binding of an Isoform-Selective Anti-TGFβ1 Antibody. *MAbs* **2018**, *10*, 444–452, doi:10.1080/19420862.2018.1426421.
37. Reid, J.; Zamuner, S.; Edwards, K.; Rumley, S.-A.; Nevin, K.; Feeney, M.; Zecchin, C.; Fernando, D.; Wisniacki, N. In Vivo Affinity and Target Engagement in Skin and Blood in a First-Time-in-Human Study of an Anti-Oncostatin M Monoclonal Antibody. *Br J Clin Pharmacol* **2018**, *84*, 2280–2291, doi:10.1111/bcp.13669.
38. Asengeprast, a Novel G-Protein Coupled Receptor 68 Antagonist, Reverses Clinically Relevant Inflammatory and Fibrotic Pathways in Chronic Kidney Disease Available online: https://sciety.org/articles/activity/10.1101/2025.02.21.25322641 (accessed on 15 October 2025).
39. Hussain, M.; Le Moulec, S.; Gimmi, C.; Bruns, R.; Straub, J.; Miller, K.; PERSEUS Study Group Differential Effect on Bone Lesions of Targeting Integrins: Randomized Phase II Trial of Abituzumab in Patients with Metastatic Castration-Resistant Prostate Cancer. *Clin Cancer Res* **2016**, *22*, 3192–3200, doi:10.1158/1078-0432.CCR-15-2512.
40. Sandner, P.; Stasch, J.P. Anti-Fibrotic Effects of Soluble Guanylate Cyclase Stimulators and Activators: A Review of the Preclinical Evidence. *Respir Med* **2017**, *122 Suppl 1*, S1–S9, doi:10.1016/j.rmed.2016.08.022.
41. Maher, T.M.; Ford, P.; Brown, K.K.; Costabel, U.; Cottin, V.; Danoff, S.K.; Groenveld, I.; Helmer, E.; Jenkins, R.G.; Milner, J.; et al. Ziritaxestat, a Novel Autotaxin Inhibitor, and Lung Function in Idiopathic Pulmonary Fibrosis: The ISABELA 1 and 2 Randomized Clinical Trials. *JAMA* **2023**, *329*, 1567–1578, doi:10.1001/jama.2023.5355.
42. Iqbal, N.; Iqbal, N. Imatinib: A Breakthrough of Targeted Therapy in Cancer. *Chemother Res Pract* **2014**, *2014*, 357027, doi:10.1155/2014/357027.
43. Chen, D.; Frezza, M.; Schmitt, S.; Kanwar, J.; Dou, Q.P. Bortezomib as the First Proteasome Inhibitor Anticancer Drug: Current Status and Future Perspectives. *Curr Cancer Drug Targets* **2011**, *11*, 239–253, doi:10.2174/156800911794519752.
44. Nagelkerke, S.Q.; Kuijpers, T.W. Immunomodulation by IVIg and the Role of Fc-Gamma Receptors: Classic Mechanisms of Action after All? *Front Immunol* **2014**, *5*, 674, doi:10.3389/fimmu.2014.00674.
